# Adjuvant-induced Human Monocyte Secretome Profiles Reveal Adjuvant- and Age-specific Protein Signatures*[Fn FN2]

**DOI:** 10.1074/mcp.M115.055541

**Published:** 2016-03-01

**Authors:** Djin-Ye Oh, David J. Dowling, Saima Ahmed, Hyungwon Choi, Spencer Brightman, Ilana Bergelson, Sebastian T. Berger, John F. Sauld, Matthew Pettengill, Alvin T. Kho, Henry J. Pollack, Hanno Steen, Ofer Levy

**Affiliations:** From the ‡Department of Medicine, Division of Infectious Diseases, Boston Children's Hospital and; §Harvard Medical School, Boston, Massachusetts;; ¶Division of Pediatric Infectious Diseases, New York University Medical School, New York;; ‖Department of Pathology, Boston Children's Hospital and Harvard Medical School, Boston, Massachusetts;; **Saw Swee Hock School of Public Health, National University of Singapore, Singapore;; ‡‡Children's Hospital Informatics Program, Boston Children's Hospital and Harvard Medical School, Boston, Massachusetts;; ^a^Precision Vaccines Program, Division of Infectious Diseases, Boston Children's Hospital, Boston, Massachusetts

## Abstract

Adjuvants boost vaccine responses, enhancing protective immunity against infections that are most common among the very young. Many adjuvants activate innate immunity, some via *Toll*-Like Receptors (TLRs), whose activities varies with age. Accordingly, characterization of age-specific adjuvant-induced immune responses may inform rational adjuvant design targeting vulnerable populations. In this study, we employed proteomics to characterize the adjuvant-induced changes of secretomes from human newborn and adult monocytes in response to Alum, the most commonly used adjuvant in licensed vaccines; Monophosphoryl Lipid A (MPLA), a TLR4-activating adjuvant component of a licensed Human Papilloma Virus vaccine; and R848 an imidazoquinoline TLR7/8 agonist that is a candidate adjuvant for early life vaccines. Monocytes were incubated *in vitro* for 24 h with vehicle, Alum, MPLA, or R848 and supernatants collected for proteomic analysis employing liquid chromatography-mass spectrometry (LC-MS) (data available via ProteomeXchange, ID PXD003534). 1894 non-redundant proteins were identified, of which ∼30 - 40% were common to all treatment conditions and ∼5% were treatment-specific. Adjuvant-stimulated secretome profiles, as identified by cluster analyses of over-represented proteins, varied with age and adjuvant type. Adjuvants, especially Alum, activated multiple innate immune pathways as assessed by functional enrichment analyses. Release of lactoferrin, pentraxin 3, and matrix metalloproteinase-9 was confirmed in newborn and adult whole blood and blood monocytes stimulated with adjuvants alone or adjuvanted licensed vaccines with distinct clinical reactogenicity profiles. MPLA-induced adult monocyte secretome profiles correlated *in silico* with transcriptome profiles induced in adults immunized with the MPLA-adjuvanted RTS,S malaria vaccine (Mosquirix™). Overall, adjuvants such as Alum, MPLA and R848 give rise to distinct and age-specific monocyte secretome profiles, paralleling responses to adjuvant-containing vaccines *in vivo*. Age-specific *in vitro* modeling coupled with proteomics may provide fresh insight into the ontogeny of adjuvant action thereby informing targeted adjuvanted vaccine development for distinct age groups.

Infections cause enormous morbidity and mortality worldwide, especially among those at the extremes of life, newborns and the elderly. Immunization is one of the most cost effective interventions to reduce infectious diseases but the efficacy of many vaccines varies with age with suboptimal vaccine responses especially common in the young and elderly, who display distinct immune system function (1, 2). Adjuvants enhance the quantity and shape the quality of vaccine-induced immune protection. Most current adjuvanted vaccines have been developed empirically, formulating antigen with adjuvants, such as aluminum salts or oil-in water emulsions, whose mode of action, especially in different age groups, is incompletely elucidated (3, 4). One approach to increase vaccine immunogenicity in these vulnerable populations is to use and develop adjuvants that are particularly effective in overcoming age-specific limitations in immune system function (5–7). Characterization of adjuvant action in different age groups may inform more rational adjuvant design.

Adjuvant activity can be age-dependent (8, 9). Responses to Alum, the most commonly used vaccine adjuvant (10, 11), vary with age (8, 9). When tested in whole blood cultures *in vitro*, Alum induces greater cytokine production (*e.g.* IL-1β and CXCL8) from newborn than adult leukocytes (12). Moreover, the age at which Alum-adjuvanted Pneumococcal conjugate vaccine is given affects subsequent innate immune polarization such that subsequent *Toll*-Like Receptor (TLR)^1^-mediated cytokine responses are altered (13). Given the extensive use of Alum as an adjuvant (10, 11), it is important to better characterize its age-specific actions.

The characterization of TLRs and other pattern recognition receptors (PRRs) linking innate and adaptive immunity has informed rational adjuvant design, with multiple PRR agonists in development as vaccine adjuvants (6, 14). The first well-defined TLR agonist (TLRA) to be incorporated into a licensed vaccine was monophosphoryl lipid A (MPLA; TLR4A), a low-toxicity lipopolysaccharide (LPS) derivative that is a component of the licensed human papilloma virus (HPV) and certain hepatitis B vaccine (HBV) formulations (15–17). Agonists of TLR3, -4, -5, -7/8 and -9 are currently in preclinical and clinical development, but TLR-mediated immune responses are often distinct at the extremes of age (2, 14). For example, when compared with their adult counterparts *in vitro*, human newborn and infant leukocytes demonstrate reduced Th1-polarizing cytokine induction in response to most TLRAs, including the TLR4As LPS and MPLA (2). For this reason, we choose to study human monocytes, leukocytes with antigen-presenting properties that are key players in the vaccine-induced immune response (18, 19). Adjuvants act via PRRs that mediate monocyte activation, triggering subsequent immune responses *in vitro* (18, 20, 21) and *in vivo* (22).

Rational adjuvant design aims to maximize a vaccine's safety and efficacy profiles, inducing an optimal protective immune response without provoking short- or long-term adverse effects. Little is known regarding the global molecular impact of adjuvants and adjuvanted vaccines in early life (23). To this end, a detailed and comprehensive understanding of adjuvant action in special target populations as compared with healthy middle aged adults is of scientific and translational interest (1). For example, TLR8As such as the imidazoquinoline R848 (TLR7/8A), can induce adult-like Th cytokine induction and co-stimulatory molecule expression in neonatal leukocytes suggesting they may be promising candidate neonatal vaccine adjuvants (18, 24–26). For novel adjuvants, a practical approach to characterize adjuvant-induced responses employs *in vitro* human cell systems that model the immune response (21, 26–28), and may inform subsequent adjuvant selection for *in vivo* studies.

Systems-scale transcriptomic approaches have increasingly been employed to examine the global immune response to adjuvanted vaccines, raising novel hypotheses regarding mechanisms of adjuvant action (29, 30) and revealing vaccine-specific molecular signatures that may predict vaccine responses (31–34). However, alterations observed in the global gene expression profiles may not necessarily correspond closely to adjuvant-induced changes on protein level (35, 36). Moreover, to date these studies have not taken into account age, even though the majorities of infection-induced mortality and of global immunization schedules are focused on newborns and infants.

The study of protein secretion in *in vitro* systems has largely depended on targeted detection methods, including measurement of cytokine production, limited by the specificity and availability of antibodies. Quantitative high-resolution mass spectrometry has been recently applied to characterize the secreted proteomes (secretomes) of murine antigen-presenting cells in a comprehensive manner (37). To gain fresh insights into the potential age-specific action of established and candidate adjuvants, we assessed the effect of treatment with Alum or the TLRAs MPLA or R848 on the secretomes of primary human monocytes. We found that (1) adjuvant-induced monocyte secretomes varied by adjuvant and age, (2) that adjuvants induce distinct innate immune pathways, (3) that protein biomarkers defined by this approach were confirmed in adjuvant and licensed adjuvanted vaccine-stimulated whole blood assays *in vitro* as well as in (4) publicly available data sets of individuals receiving a novel adjuvanted vaccine *in vivo*. Our observations provide fresh insight into adjuvant action and suggest a novel paradigm for characterizing age-specific adjuvant effects *in vitro* in relation to their effects *in vivo*.

## EXPERIMENTAL PROCEDURES

### 

#### 

##### Ethics Statement

All experiments were conducted in accordance with relevant institutional and national guidelines, regulations and approvals. Cord blood samples were collected and de-identified with approval from the Ethics Committee of The Brigham & Women's Hospital, Boston, MA (protocol number 2000-P-000117) and Beth Israel Deaconess Medical Center Boston, MA (protocol number 2011P-000118). Blood from adult donors was obtained after written informed consent with approval from the Ethics Committee of Boston Children's Hospital; Boston, MA (protocol number X07–05-0223).

##### TLR Agonists, Adjuvants, Licensed Vaccines, and Assay Reagents

TLRAs included MPLA (TLR4), R848 (TLR7/8), and were obtained from InvivoGen (San Diego, CA). Adju-Phos (Al (PO_4_)_3_) was from The Statens Serum Institut (Copenhagen, Denmark) (Table I). TLRAs (other than MPLA) were verified to be endotoxin-free (< 1 EU/ml) as measured by the *Limulus* amebocyte lysate (LAL) assay per the manufacturer's instructions (Charles River; Wilmington, MA). Licensed adjuvanted vaccines were obtained from manufacturers as listed in Table II.

##### Monocyte Isolation and In Vitro Stimulation Assay

Peripheral blood was collected from healthy adult volunteers, whereas human newborn cord blood was collected immediately after Cesarean section delivery of the placenta. Placentas from HIV-positive mothers were excluded. Blood was anti-coagulated with 15–20 units/ml pyrogen-free sodium heparin (American Pharmaceutical Partners, Inc.; Schaumberg, IL), kept at room temperature and processed within 4 h of collection. Cord blood mononuclear cells (CBMC) or peripheral blood mononuclear cells (PBMC) were obtained after centrifugation over Ficoll-Hypaque gradients (Ficoll-Paque PREMIUM, GE Healthcare; Waukesha, WI). Monocytes were isolated from mononuclear cell fractions by positive selection with magnetic microbeads according to the manufacturer's instructions (Miltenyi Biotec; Auburn, CA) using CD14 as a pan-marker for monocytes (38). Monocyte preparations were verified to be > 95% CD14 positive as assessed by flow cytometry for CD14 with fluorophore-labeled antibodies (FITC, BD Biosciences) as previously described (39). For secretomic studies, isolated monocytes were cultured in tissue culture dishes at 4 × 10^6^ cells/ml in high glucose Dulbecco's Modified Eagle Medium (DMEM, Life Technologies; Grand Island, NY) supplemented with non-essential amino acids solution (Life Technologies) in the presence of sterile medium (negative control), Alum (5 μg/ml), MPLA (100, 1000 ng/ml), or R848 (5, 50 μm). For targeted protein confirmation assays, monocytes were cultured in 96-well tissue round bottom culture dishes at 1 × 10^6^ cells/ml in either RPMI supplemented with 10% platelet poor plasma. Monocytes were then incubated at 37 °C, 5% CO_2_ with or without adjuvants for 18 h.

##### Supernatant Sample Preparation for Secretome Analysis

Conditioned culture media samples were collected after 18 h of incubation, centrifuged for 5 min at 500 × *g*, 4 °C and the resulting supernatant transferred to a new micro-tube before storage at −80 °C until further use. Upon thawing, proteins were precipitated from the supernatants using trichloroacetic acid and prepared for LC-MS analysis. Concentrated protein samples were reduced with dithiothreitol (DTT) and alkylated with acrylamide before they were applied to 12% gradient gels for sodium dodecyl sulfate polyacrylamide gel electrophoresis (SDS-PAGE) (NuPage; Invitrogen, NY). After staining with Coomassie Blue (SimplyBlue Safestain; Invitrogen), the gel lanes were excised and subjected to in-gel tryptic digestion. Gel pieces were destained in ammonium bicarbonate and acetonitrile, dehydrated in acetonitrile, and dried using a SpeedVac. Finally, samples were digested overnight with 12 ng/μl trypsin at room temperature.

##### LC-MS Analysis

Peptides were analyzed on a nanoflow ultrahigh-performance liquid chromatography (UPLC) system (Eksigent/Sciex; Framingham, MA) hyphenated with a quadrupole-Orbitrap mass spectrometer (Q Exactive; Thermo Scientific; San Jose, CA). Peptides were loaded onto an in-house packed 100 μm i.d. × 15 cm C18 column (Magic C18, 5 μm, 200Å; Michrom Bioresource; Auburn, CA) and separated at 400 nl/min (linear gradients from 5 to 35% acetonitrile in 0.2% formic acid). Full MS scans were obtained in an Orbitrap mass analyzer over m/z 350–2000 range with resolution 70,000 (m/z 200) and a target value of 3 × 10^6^. The 10 most intense peaks (charge state ≥ 2) were fragmented in the higher-energy collisional dissociation (HCD) collision cell at normalized collision energy of 27%, and tandem mass spectrum was acquired in the Orbitrap mass analyzer with resolution 17500 at m/z 200 and a target value of 2 × 10^5^. The ion selection threshold was 1.7 × 10^3^, the maximum allowed ion accumulation times were 20 millisecond (ms) for full MS scans and 120 ms for tandem mass spectrum. For each MS run, RAW files, generated using XCalibur software (version 2.2; Thermo Scientific), were converted to Mascot Generic Files (MGF) through an in-house script, extracting the 200 most intense fragment ions for each MS/MS spectrum. The mass spectrometry proteomics data have been deposited to the ProteomeXchange Consortium (40) via the PRIDE partner repository with the data set identifier PXD003534 and 10.6019/PXD003534.

##### Protein Identification

All peak list files (MGFs) were analyzed using the Mascot (Matrix Science; London, UK; version 2.3.02) and X!Tandem search engines (The GPM, thegpm.org; version CYCLONE (2010.12.01.1)). Mascot was configured to search the Uniprot human sequence database (35,806 entries as of May 2011), assuming the digestion enzyme trypsin with one missed cleavage allowed. Searches were conducted with fixed modification for carbamidomethyl; variable methionine oxidation and variable pyroGlu formation (from N-terminal Glu and Gln); monoisotopic mass with peptide precursor mass tolerance of 10 parts per million (ppm); MS/MS ion mass tolerance of 0.02 Dalton (Da). X!Tandem was set up to search a subset of Uniprot human sequence database, also assuming trypsin, allowing for up to two missed cleavages; a fragment ion mass tolerance of 0.02 Da and a parent ion tolerance of 10.0 ppm; Carbamidomethyl of cysteine was specified as a fixed, and Glu->pyro-Glu of the N terminus, Gln->pyro-Glu of the N terminus, ammonia-loss of the N terminus, and methionine oxidation were specified in X!Tandem as variable modifications. Scaffold (Proteome Software Inc., Portland, OR; version 4.2.1) was used to combine the MS/MS-based peptide and protein identifications. Peptide identifications were accepted at a false discovery rate (FDR) < 1%. Peptide probabilities from X!Tandem were assigned by the Scaffold Local FDR algorithm. Protein identifications were accepted if they could be established at > 95% probability to achieve an FDR <1% and contained at least one identified peptide. Of note: for several downstream data analyses steps, only proteins identified by 2 or more peptides were considered (see below). Protein probabilities were assigned by the Protein Prophet algorithm (41). Proteins that contained similar peptides and could not be differentiated based on MS/MS analysis alone were grouped to satisfy the principles of parsimony. Proteins sharing significant peptide evidence were grouped into clusters. Next, a Scaffold output file was generated, containing exclusive unique spectral counts (termed raw spectral counts hereafter) for each identified protein per biological sample.

##### Bioinformatics and Hierarchical Cluster Analysis

Proteomic data underwent hierarchical cluster analysis, using PermutMatrix, a software packet originally designed for gene expression analysis (http://www.lirmm.fr/caraux/PermutMatrix/EN/index.html) (42), that has subsequently been validated for proteomics (43, 44). Briefly, relative label-free protein quantification analysis was performed based on unique spectral counts (SpCs), which were obtained using the Scaffold 4.2.1 software. Not all proteins were identified in all samples. For visualization, comparison and data analysis purposes, proteins with missing SpC values in one sample were imputed with a SpC value of 0.5 (corresponding to each data set's half minimum SpC), thus avoiding division by zero and preventing overestimation of fold changes. For each sample, raw SpC were normalized by multiplication with the ratio of maximum total SpC and the sample's total SpC. Fold change was calculated by dividing the normalized SpC from each treated condition with that of the corresponding untreated adult or newborn condition. Cluster analysis was restricted to proteins identified by ≥2 spectra and in at least two biological samples, which showed an adjuvant-induced fold change ≥2 in more than half of the biological replicates of the same treatment condition. For each protein, average log2-transformed fold changes observed under one treatment condition were imported into PermutMatrix and Z score-transformed for normalization. Then two-way clustering analysis was carried out using the Pearson and Ward's minimum variance methods for distance and aggregation, resulting in dendograms that display treatment conditions on the horizontal and protein names on the vertical axis.

##### Label-free Protein Quantification and Analysis of Differential Protein Expression

Protein expression in differentially stimulated primary human monocytes was compared based on length-normalized SpC, which were determined using the Scaffold 4.2.1 software (as outlined above). Based on results of a small pilot study (*n* = 3) using multiple replicates (∼ 3) of the adjuvants tested, which indicated that singlet samples were representative, one Scaffold file was generated containing all SpCs for each of the 6 individual adult and 7 individual newborn monocyte singlet samples treated with different adjuvants. Multiple isoforms underwent quantification only if they were classified as individual entries (with distinct gene names) by Scaffold. Proteins enriched after adjuvant treatment but not medium treatment were identified employing a customized version of the model-based statistical analysis tool QSpec based on SpC, using the count data model with paired design option and normalization by the total number of SpC in each sample replicate (45). Proteins with QSpec's new significance score (Z-statistic) above the threshold associated with 5% FDR underwent further evaluation (46).

##### Content Analysis

Secretome data were further analyzed using Ingenuity Pathway Analysis (IPA) (Ingenuity® Systems, www.ingenuity.com). Canonical pathways analysis identified the pathways from the IPA library of canonical pathways that were most significant to each secretome induced in neonates and adults by Alum, MPLA and R848. Identified proteins that i) met the FDR cutoff of 5% and ii) were associated with a canonical pathway in the Ingenuity Knowledge Base were considered for the analysis. The significance of the association between the data set and the canonical pathway was measured using Fisher's exact test, which calculates the probability that the overlap between the genes in the data set and the canonical pathway is explained by chance alone. The Odds Ratio for the overlap was calculated for each significantly up-regulated pathway.

##### Whole Blood Assay (WBA)

Adjuvant and vaccine activity in whole blood (WB) was assessed by adapting previously described methodologies (24, 47). Briefly, heparinized neonatal cord blood or adult whole blood, obtained from different study participants than those providing blood for the monocyte stimulation assays, was mixed 1:1 with sterile pre-warmed (37 °C) RPMI 1640 medium (Invitrogen) and cultured in 96-well U-bottom plates (Becton Dickinson, Franklin Lakes, NJ) in the presence of adjuvants (Alum [0.5, 5 and 50 μg/ml]; MPLA [1, 10, 100 and 1000 ng/ml]; R848 [0.5, 5 and 50 μm]) or the vaccines listed in Table II (final dilutions 1:10, 1:100 and 1:1000). Suspensions containing 200 μl/well were gently mixed several times by pipetting and incubated for 6 h, 37 °C in a humidified incubator at 5% CO_2_. After culture, plates were centrifuged at 500 × *g* and 110–150 μl of supernatant was removed by pipetting without disturbing the cell pellet. Supernatants were assayed by ELISA to quantify concentrations of Lactoferrin (LTF; Hycult Biotech, Uden, The Netherlands), Pentraxin 3 (PTX-3; R&D Systems, Minneapolis, MN), matrix metalloproteinase 9 (MMP-9; R&D Systems), TNF (BD Biosciences), IL-1β (eBiosciences, San Diego, CA) and by competitive EIA for Prostaglandin E_2_ (PGE_2_; Cayman Chemical, Ann Arbor, MI). Adenosine deaminase-2 (ADA2) activity in whole blood assay supernatants was determined using a chromogenic enzyme activity assay (Diazyme Laboratories, Poway, CA), run in duplicate in the presence of erythro-9-(2-hydroxy-3-nonyl)adenine (EHNA; 20 μm) to inhibit ADA-1 activity. ADA2 is not EHNA-sensitive; thus, activity in the presence of EHNA reflects ADA2 activity (48).

##### Extrapolation of the Adjuvant-Induced Monocyte Secretome Data Set to an Adjuvanted-Vaccine-Induced PBMC Transcriptome Data Set

Each protein present in the adjuvant-induced monocyte secretomes was quantified based on spectral count data, using QSpec as described above. Changes relative to the secretomes of medium-treated controls were considered statistically significant based on Z-statistics corresponding to an FDR < 5%. PBMC transcriptome profiles (49) of 24 adults vaccinated with the MPLA adjuvant-containing Mosquirix™ Malaria vaccine RTS,S/AS01 (dispersed lipid vesicles containing MPLA and saponin) or the similar formulation RTS,S/AS02 (an oil-in-water preparation containing MPLA and saponin), measured at four different time points (T1 = 0 h, T2 = 24 h, T3 = 48 h, T4 = 14 days post third vaccine dose), and publicly available at http://www.ncbi.nlm.nih.gov/geo/query/acc.cgi?acc=GSE18323, were analyzed for changes in gene expression after immunization (50). GSE18323 was Robust Multi-array Average (RMA) quantile normalized (51) for all 11 vaccine-protected and 13 non-protected study participant transcriptome profiles at 4 time points (T1 - T4). Paired (by participant) nonparametric Wilcoxon signed-rank test was applied at *p* < 0.05 significance threshold per microarray probe (*i.e.* gene) between T2 *versus* T1 time points across the non-protected participant subset, the protected participant subset, and combined non-protected and protected participant subsets respectively. A similar analysis was conducted for T3 *versus* T1, T4 *versus* T1. Each microarray probe was mapped to a gene identified by its Entrez Gene ID. For a gene with > 1 microarray probe, we used the microarray probe with the smallest *p* value from the paired differential analysis above as the unique representative for that gene. Each gene/Entrez Gene ID was matched to corresponding protein accession numbers of the secretome data set via ftp://ftp.ncbi.nih.gov/gene/DATA/gene2accession.gz version 2015 May 19. Molecules present in both *in vitro* monocyte secretome and *in vivo* PBMC transcriptome data sets were assessed for the direction of their change (*versus* the unstimulated condition and the T1 time point, respectively) and its statistical significance. The set of proteins displaying significant changes in the MPLA-induced adult secretome, and each set of genes displaying significant changes in one of the three time comparisons of GSE18323, underwent gene ontology (GO) analysis using https://david.ncifcrf.gov/with EASE score (alpha) of 0.05 to call significance. The resulting significant GO terms were evaluated with respect to their co-occurrence between MPLA secretome and each of the three transcriptome data sets in GSE18323.

##### Experimental Design and Statistical Rationale

The total numbers of biological samples analyzed for the monocyte stimulation assay are 6 for adult and 7 for newborn, whereas the numbers of biological samples analyzed for WBA was *n* = 4 - 8. Data used to extrapolate adjuvant-induced secretome to adjuvanted-induced transcriptome, was from 24 adult study participants. Flow cytometry analysis employed a MoFlo Legacy cytometer (DakoCytomation; Fort Collins, CO) or FACSFortessa (BD Biosciences) cytometer and was analyzed using FlowJo software (Tree Star). Graphic output was generated using JMP v. 11.0 (SAS, Cary NC) and Prism v. 6.0c (GraphPad Software). For WBA confirmation assays, statistical significance was determined using two-tailed student *t* tests comparing stimulated samples to unstimulated controls. Results were considered statistically significant at *p* < 0.05. To assess differential expression of Recombivax (HBV)- *versus* EasyFive (DTwP-Hep B-Hib)- induced protein fold changes, the Wilcoxon rank-sum test was used, combining all dosages of each vaccine. This is a nonparametric test of the null hypothesis (that the two groups of fold changes result from the same treatment condition) against an alternative hypothesis, where the null hypothesis was rejected for *p* values less than 0.05.

## RESULTS

### 

#### 

##### Protein Identification by LC-MS from Monocyte Cultures

**Fig. 1.**
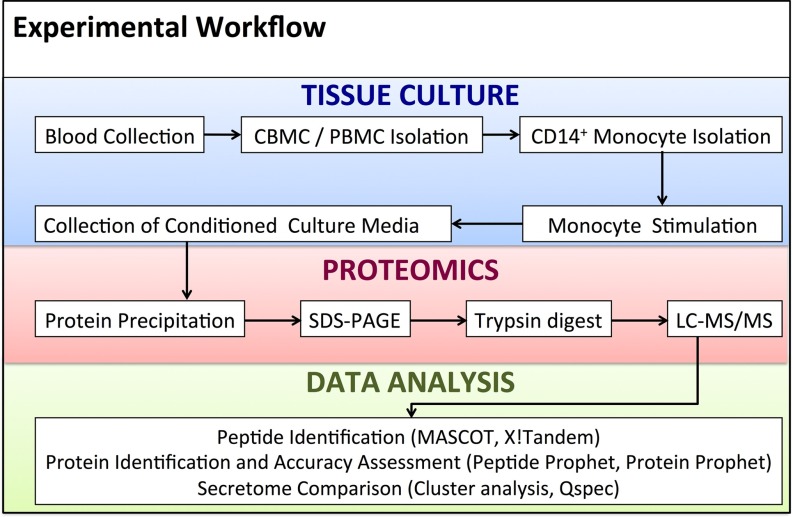
**Protocol employed for secretome analysis of adjuvant-stimulated primary human newborn and adult monocytes.** Human monocytes, isolated as described in Methods, were cultured under serum-free conditions for 18 h in the presence of sterile buffer (control), Alum [5 μg/ml], MPLA [100 ng/ml], and R848 [5 μm]. Supernatant proteins were precipitated prior to SDS-PAGE and trypsin digestion. The resulting peptide mixtures were subjected to LC-MS/MS on a QE mass spectrometer (3 h gradient). Several levels of data analysis were undertaken for protein identification and secretome comparison.

Culture supernatants from neonatal and adult human monocytes treated with medium alone (control), aluminum phosphate (Alum), MPLA or R848 (Table I) were analyzed by LC-MS using a nanoflow HPLC system hyphenated with a hybrid quadrupole/Orbitrap mass spectrometer (summarized in Fig. 1). Using the MASCOT and X!Tandem search engines, a total of 1894 nonredundant proteins was identified. The number of proteins identified in supernatants from differentially treated cells ranged from 829 to 1184 for the neonatal, and from 832 to 1406 for the adult samples, respectively. supplemental Table S1 gives an overview of these proteins, which were identified in seven biological replicates (*i.e.* six paired adult and newborn samples, and one unpaired newborn sample) combined, with a raw spectral count ≥ 1 and a protein FDR < 1%.

**Table I:** Characteristics of adjuvants used in this study

Name	Mechanism of immune activation	Current use
AlPO	NLRP3 Inflammasome, IRF3	Component of licensed vaccines, including HBV, PCV
MPLA	TLR4 activation	Approved for HPV vaccine (Cervarix)
R848	TLR7/8 activation, NLR activation	R848 and additional TLR7/8 activating molecules are in preclinical development as vaccine adjuvants

MPLA; monophospholipid A, TLR; Toll-like receptor, HBV; Hepatitis B Vaccine, PCV; Pneumococcal conjugate vaccine, NLR; NOD-like receptor.

##### Protein Overlap in Supernatants from Differentially Stimulated Monocytes Samples and Hierarchical Clustering

**Fig. 2.**
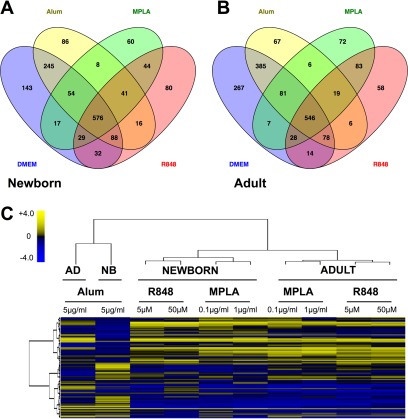
**Monocyte secretomes are adjuvant- and age-specific.** *A*, *B*, Distribution of proteins determined by LC-MS/MS in the supernatants of differentially treated newborn and adult monocytes. *C*, Cluster analysis of adjuvant-induced human monocyte secretomes indicates age- and adjuvant-specific differences. Proteins identified in the supernatants from CD14+ monocytes cultured in the presence of Alum (5 μg/ml), MPLA (100 - 1000 ng/ml), or R848 (5 - 50 μm) are shown along the *y* axis and treatment conditions along the *x* axis. Neonatal and Adult secretomes clustered separately. The R848-induced secretomes clustered together with the MPLA-secretome and are distinct from the Alum-induced secretome. Color codes reflect the Z scores of the log2-transformed average changes of normalized spectral count (SpC) relative to the untreated control sample. Data are representative of proteins identified in newborn (*n* = 7) and adult (*n* = 6) biological replicates, of which six included paired newborn/adult samples.

Of the 1519 and 1717 non-redundant proteins identified for the newborn and the adult samples, respectively, 617 (41%) and 565 (33%) were common to all adjuvant treatment conditions and an average of 75 (5%) and 67 (4%) proteins were unique to each (Fig. 2*A*, 2*B*). Proteins detected at a spectral count of 1 in a single sample of only one condition were excluded from all subsequent analyses. As a first approach to compare secreted proteomes (secretomes) resulting from differential stimulation of newborn and adult human monocytes, we analyzed proteomic data sets using unsupervised two-way hierarchical clustering analysis, based on each protein's average fold change per treatment condition. A heat map was generated to visualize the results (Fig. 2*C*). Alum-induced newborn and adult monocyte secretomes demonstrated the most marked differences, exceeding the degree of variation observed between newborn and adult for each of the TLRAs. Age alone appeared to exert an effect on TLRA-induced secretomes, resulting in distinct newborn and adult clusters. Furthermore, both the TLRAs MPLA (TLR4) and R848 (TLR8), induced similar secretome clusters by age that were highly distinct from those induced by Alum (Fig. 2*C*).

##### Differential Expression of Proteins in Secretomes from Monocytes Treated with Alum, MPLA and R848

**Fig. 3.**
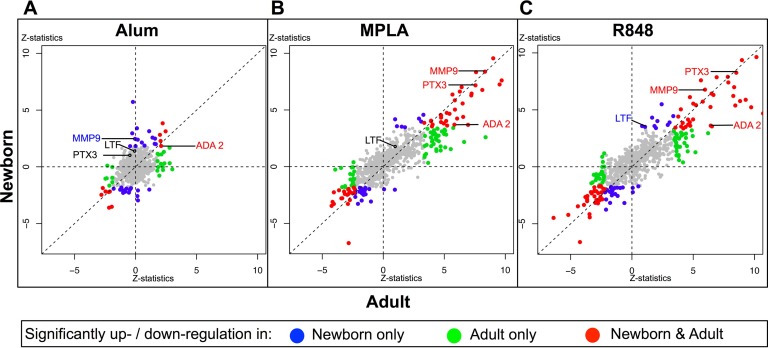
**Alum-, MPLA-, and R848-induced human monocyte secretomes in newborns and adults demonstrate age-common and age-specific proteins.** Secretome proteins after stimulation of newborn (*y* axis) and adult (*x* axis) monocytes with Alum (*A*, 5 μg/ml), MPLA (*B*, 100 ng/ml) or R848 (*C*, 5 μm) are plotted by Z statistic values, reflecting the average change of normalized spectral counts in relation to sterile buffer treatment, taking inter-individual variability into account. Each circle represents one protein. Highlighted in colors are proteins that demonstrated statistically significant enrichment after adjuvant treatment in neonates exclusively (blue), adults exclusively (green) or both neonates and adults (red). Proteins selected for targeted confirmation based on reproducibility of the observed fold change across multiple specimens, novelty and commercial assay availability are labeled. Data represents proteins identified in biological replicates for newborn (*n* = 7) and adult (*n* = 6) monocyte secretomes.

Next, the proteins differentially expressed between the un-stimulated and the adjuvant treatment conditions were further examined. For each treatment condition, proteins that were significantly enriched relative to the un-stimulated controls were identified using QSpec, a Bayesian statistical framework specifically designed to handle discrete, spectral count-based data (45). This software was customized for the purpose of this study to facilitate pairwise analysis within each stimulation experiment, aggregating evidence across multiple experiments. Fig. 3 visualizes, for newborns and adults, adjuvant-induced increases or decreases in protein expression levels, highlighting those changed significantly at an FDR < 5%. The distributions of the proteins in the three different treatments recapitulate the finding described in Fig. 2*C* that the MPLA and R848 treatments elicit much more similar responses than the Alum treatment. For each adjuvant, these proteins can be grouped into those that are significantly up-, or down- regulated in neonates exclusively (blue circle), adults exclusively (green) or both neonates and adults (red), at an FDR < 0.05. Among the total 218 significantly changed proteins, 115 were significantly enriched in response to at least one adjuvant in the neonatal secretomes (66 proteins/blue circles, Fig. 3*A*–3*C*) and/or the adult secretomes (100 proteins/green, Fig. 3*A*–3*C*). Further details on significantly enriched proteins are provided in the supplemental section online, which includes information on whether enriched proteins are induced by more than one adjuvant (supplemental Fig. S1, for which an interactive version is accessible at http://adjuvant-secretomes.herokuapp.com/), as well as accession numbers, predicted secretion mechanisms, fold changes, Z statistics and FDR values for each protein (supplemental Table S2, supplemental Table S3).

##### Pathway Analysis

The results of IPA pathway analyses are depicted in Table III. Pathways represented in the Alum-induced secretomes differed substantially between newborn and adult (Table III and supplemental Fig. S2*A*). The neonatal Alum-induced secretome was significantly enriched with proteins belonging to innate immune pathways, including granulocyte adhesion signaling, leukocyte extravasation/diapedesis, crosstalk between dendritic and NK T cells, and Interleukin-8 (CXCL-8) signaling. In contrast, many of thesepathways, such as DC/NKT cell crosstalk, T helper cell differentiation and communication of innate and adaptive immune cells were underrepresented in the adult Alum-induced secretome. The Alum-induced adult secretome, on the other hand, included unique proteins of the actin cytoskeleton pathway, possibly relating to cell degradation (52) (Table III and supplemental Fig. S2*A*). Multiple pathways represented in the TLRA-induced monocyte secretomes (Table III and supplemental Fig. S2*B*–S2*C*), such as Acute Phase Response Signaling, LXR/RXR Activation and Granulocyte Adhesion/Diapedesis Signaling, are relevant to the immune response and correspond to some of those present in the neonatal Alum secretome. In contrast to the highly divergent Alum response by age, greater similarity was observed between newborn and adult secretomes induced by the two investigated TLRAs.

##### Selective Verification of Secretomics Results using in vitro Monocyte and Whole Blood Assays

**Fig. 4.**
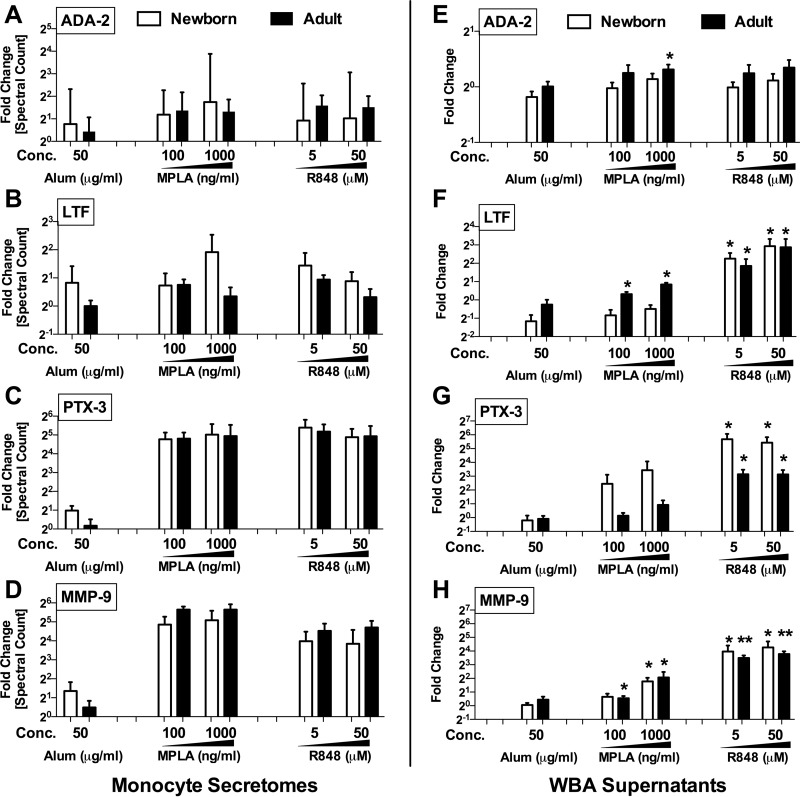
**Targeted confirmation of proteins identified by monocyte secretomics in human whole blood assay.** To confirm the adjuvant-induced release of adenosine deaminase 2 (ADA-2), lactoferrin (LTF), pentraxin 3 (PTX-3) and matrix metalloproteinase (MMP-9) observed in monocyte secretomes (*A–D*, Alum: 50 μg/ml, MPLA: 100 and 1000 ng/ml, R848: 5 and 50 μm), human neonatal and adult blood was stimulated with vehicle (RPMI), Alum (0.5–50 μg/ml), MPLA (TLR4; 10–1000 ng/ml), or R848 (TLR7/8; 0.5–50 μm). *E–H*, Supernatants were assayed for ADA-2 activity (*E,* kinetic assay) as well as LTF, PTX-3 and MMP-9 concentrations (*F–H*, ELISAs). Data represent fold change (mean ± S.E.) in stimulated samples *versus* the untreated controls (neonates and adults; *n* = 5–8/age group). □ = Newborn, ■ = Adult. *, *p* < 0.05 as determined by 2-tailed Student's *t* test.

Following secretomics analysis, we wished to confirm up-regulation of selected proteins in a different experimental system. Selection of proteins for confirmation took into account novelty, significance level and reproducibility across multiple samples as well as commercial availability of targeted quantification assays (*e.g.* ELISA). Four proteins were measured in human whole blood stimulation assays (Fig. 4*E*–4*H*; each protein's corresponding spectral counts observed in the previous monocyte secretome analysis are shown in Fig. 4*A*–4*D*): adenosine deaminase 2 (ADA-2) (Fig. 4*E*), lactoferrin (LTF) (Fig. 4*F*), pentraxin 3 (PTX-3) (Fig. 4*G*), and matrix metalloproteinase 9 (MMP-9) (Fig. 4*H*).

Whereas ADA-2 activity displayed only minimal changes in the WBA system, statistically significant elevations in response to adjuvant treatment of whole blood were observed for LTF, PTX-3 and MMP-9. LTF levels rose moderately after MPLA treatment of adult WB (100 ng/ml, *p* = 0.03 and 1000 ng/ml, *p* = 0.04), and over twofold after 50 μm R848 treatment of both newborn (*p* = 0.001) and adult WB to (*p* = 0.01). Significant PTX-3 increases were induced in newborn WB by MPLA (10 ng/ml, *p* = 0.01) and R848 (all concentrations, *p* = 0.01), as well as R848 in adult WB (0.5 μm, *p* = 0.048; 5 μm. *p* = 0.002; 50 μm, *p* = 0.002). MMP-9 levels significantly increased in response to 100 ng/ml of MPLA in both newborn (*p* = 0.004) and adult WB (*p* = 0.02), whereas titration dependent responses were observed in newborn (all concentrations, *p* = 0.01) to as well as adult WB (0.5 μm, *p* = 0.03; 5 μm. *p* = 0.002; 50 μm, *p* < 0.001). To further confirm that targeted biomarkers are directly produced by blood monocytes, we purified CD14+ monocytes by immunomagnetic bead selection, cultured them in RPMI supplemented with 10% plasma, and stimulated them with Alum, MPLA, or R848. Supernatants were collected for subsequent ELISAs that demonstrated production of LTF (supplemental Fig. S3*A,* S3*B*), PTX-3 (supplemental Fig. S3*D,* S3*E*) and MMP-9 (supplemental Fig. S3*G,* S3*H*) in a concentration dependent manner.

##### Induction of Verified Proteins and of Cytokines by Licensed Vaccines

**Fig. 5.**
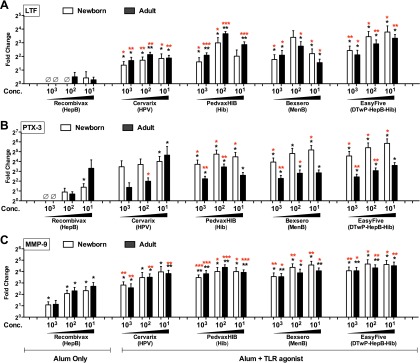
**Alum- and/or TLR agonist-adjuvanted vaccines induce distinct patterns of of LTF, PTX-3 and MMP-9 release in whole blood tested *in vitro*.** Human neonatal and adult blood was cultured in the presence of vaccines for 6 h at 37 °C prior to collection of the extracellular fluid for measurement of *A*, LTF, *B*, PTX-3 and *C*, MMP-9 by ELISA. Data are depicted as fold changes (mean ± S.E.) of *n* = 5–8 neonates and adults; asterisks denote a statistically significant difference between stimulated samples *versus* the untreated (*black stars*), or Alum only Recombivax *versus* the Alum + TLR agonist containing vaccines (*red stars*), all at equivalent volume-to-volume (v/v) treatments. *, *p* < 0.05, **, *p* < 0.01, **, *p* < 0.001 as determined by 2-tailed Student's *t* test.

**Fig. 6.**
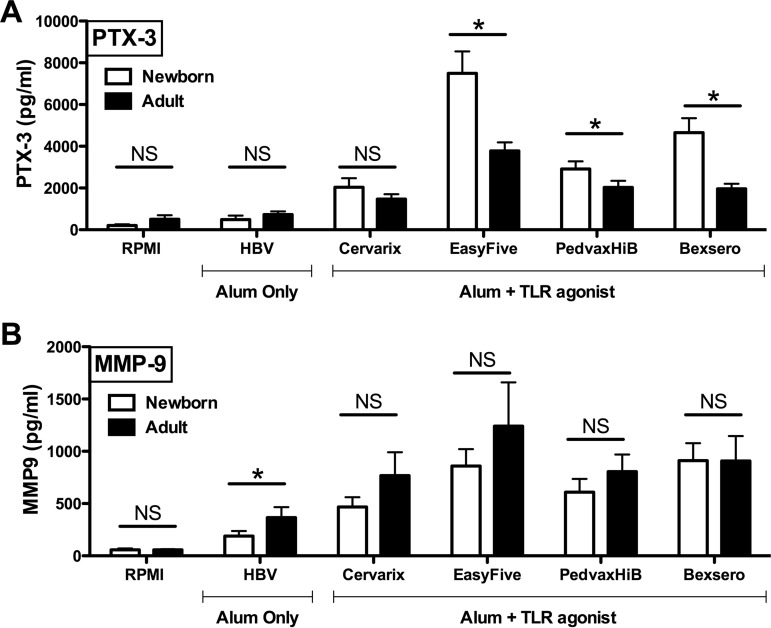
**Licensed pediatric vaccines induce age-specific PTX-3 responses in newborn whole blood *in vitro*.** Human neonatal and adult blood was cultured as outlined above. *A*, PTX-3 levels in newborn blood are significantly elevated over adult responses to (Alum + TLR agonist)- containing vaccines (*i.e.* Bexsero, PedvaxHIB and Easyfive). *B*, In contrast, adult blood demonstrates age-specific elevation in MMP-9 to the Alum-adjuvanted HBV (Recombivax), a vaccine of low reactogenicity. Data are depicted as fold changes (mean ± S.E.) of neonates and adults (*n* = 5–8/group); asterisks denote a statistically significant difference between newborn and adults, all at equivalent 1:10 volume-to-volume (v/v) treatments. *, *p* < 0.05, as determined by 2-tailed Student's *t* test.

To assess if LTF, PTX-3 and MMP-9 are also induced by adjuvanted vaccine formulations, we studied WB stimulation with five licensed vaccines that are adjuvanted with Alum alone or with Alum in combination with TLRAs (listed in Table II). When compared with the untreated control, significant elevations of LTF was observed in both newborn and adult WB treated with all of the Alum + TLRA-containing vaccines at multiple concentrations, but not with the hepatitis B vaccine (HBV) Recombivax, that is adjuvanted with Alum alone (Fig. 5*A*). LTF levels were also significantly higher when directly comparing the Alum-only Recombivax *versus* the (Alum + TLR agonist)-containing vaccines (*i.e.* Cervarix, Bexsero, PedvaxHIB and Easyfive), all at equivalent volume-to-volume (v/v) treatments concentrations. With respect to PTX-3, a similar trend was also observed. When compared at equivalent volume-to-volume (v/v) treatment concentrations, HBV (Recombivax) induced only moderate increases over the untreated control in newborn WB (*p* = 0.04 at the highest dose), whereas the (Alum + TLRA)-containing vaccines induced greater PTX-3 concentrations in both newborns and adults, in comparison to control and HBV (Fig. 5*B*). In contrast, MMP-9 was induced in a concentration- dependent manner in both newborn (*p* < 0.05) and adult (*p* < 0.05) WB by HBV (Recombivax) alone (Fig. 5*C*). Nevertheless, (Alum + TLRA)-containing vaccines induced significantly greater MMP-9 concentrations than HBV (Recombivax) in both newborns and adults (Fig. 5*C*). Interestingly, the licensed pediatric vaccine not indicated for neonatal administration *in vivo* (*i.e.* Bexsero, PedvaxHIB and Easyfive) induced age-specific PTX-3 release in newborn whole blood *in vitro* (*p* < 0.05), when compared directly to identically treated adult samples (Fig. 6*A*). In contrast, Alum-adjuvanted HBV (Recombivax), a vaccine indicated for neonatal administration and with potentially relatively lower reactogenicity rates, induced age-specific elevation of MMP-9 in adult blood (Fig. 6, 6*B*). Vaccine induced responses were also observed to be directly produced by blood monocytes (supplemental Fig. S3*C,* S3*F,* S3*I*).

**Table II:** Characteristics of the licensed vaccines used in whole blood assays

Vaccine	Trade name	Abbreviation	Manufacturer	Adjuvant
Hepatitis B	Recombivax HB	HepB	Merck	AAHS
Human Papillomavirus	Cervarix	2vHPV	GlaxoSmithKline	Al(OH)_3_, MPLA
H. influenzae type b, Meningococcal	PedvaxHIB	Hib-MenB	Merck	AAHS, OMPC
Meningococcal	Bexsero	MenB	GlaxoSmithKline	Al(OH)3, OMVs
Hepatitis B, Diphtheria, Tetanus, Pertussis, H. influenzae type b,	Easyfive	DTwP-Hep B-Hib	Panacea Biotec	AlPO, Inactivated whole cell *B. pertussis*

Table adapted from Centers for Disease Control and Prevention Epidemiology and Prevention of Vaccine-Preventable Diseases, 13th Edition. Abbreviations. AAHS; Amorphous Aluminum Hydroxyphosphate Sulfate, AlPO; Aluminum Phosphate, Al(OH)_3_; Aluminum Hydroxide, MPLA; Monophosphoryl Lipid A, OMVs; Outer Membrane Vesicles, OMPC; Outer Membrane Protein Complex.

**Table III:** Monocyte secretomes demonstrate activation of distinct canonical pathways varying by adjuvant and age. Ingenuity Pathway Analysis (IPA) was used to identify canonical pathways represented in the sets of adjuvant-induced proteins (n = 7 newborns and n = 6 adults). Proteins significantly up-regulated in the Alum-, MPLA-, and R848-induced secretomes. Shown are only those pathways represented by more than one protein in at least one of the adult/newborn datasets, with a p value < 0.05 per IPA; disease-specific pathways are excluded from the graph

Treatment	Pathways	-log10 (*p* value)	Significantly up in newborn/adult
Adult alum	Caveolar-mediated endocytosis signaling	2.56	Yes
	Integrin signaling	1.72	Yes
	Actin cytoskeleton signaling	1.66	No
	MSP-RON signaling pathway	1.31	Yes
Adult MPLA	Acute phase response signaling	1.24	Yes
	LXR/RXR Activation	8.56	Yes
	Coagulation system	6.39	No
	Granulocyte adhesion and diapedesis	6.22	Yes
	Caveolar-mediated endocytosis signaling	6.15	Yes
	Leukocyte extravasation signaling	5.70	Yes
	Complement system	4.96	Yes
	FXR/RXR Activation	4.75	Yes
	Communication between innate and adaptive immune cells	4.53	Yes
	Extrinsic prothrombin activation pathway	4.36	No
	Dendritic cell maturation	4.02	No
	Agranulocyte adhesion and diapedesis	3.93	Yes
	IL-8 Signaling	3.83	Yes
	Intrinsic prothrombin activation pathway	3.61	No
	Antigen presentation pathway	3.25	No
	Inhibition of matrix metalloproteases	3.22	No
	Virus entry via endocytic pathways	3.21	Yes
	Glucocorticoid receptor signaling	3.03	No
	Paxillin signaling	3.03	Yes
	Dermatan sulfate degradation (Metazoa)	2.78	No
	Agrin interactions at neuromuscular junction	2.50	Yes
	IL-10 Signaling	2.48	Yes
	TREM1 signaling	2.45	Yes
	TR/RXR activation	2.21	No
	Crosstalk between dendritic cells and natural killer cells	2.16	Yes
	Cytotoxic T lymphocyte-mediated apoptosis of target cells	2.09	No
	Inhibition of angiogenesis by TSP1	2.07	Yes
	IL-6 signaling	1.84	Yes
	Role of PRRs in recognition of bacteria and viruses	1.82	Yes
	Hematopoiesis from pluripotent stem cells	1.80	No
	Relaxin signaling	1.70	Yes
	Role of cytokines in mediating Communication between immune cells	1.66	Yes
	melatonin signaling	1.45	No
	Macropinocytosis signaling	1.45	Yes
	Endothelin-1 signaling	1.43	No
	Ephrin receptor signaling	1.40	No
	NF-κB activation by viruses	1.39	Yes
	Toll-like receptor signaling	1.39	Yes
	Production of nitric oxide and reactive oxygen species in macrophages	1.36	No
	Integrin-linked kinase signaling	1.35	Yes
Adult R848	Acute phase response signaling	12.8	Yes
	LXR/RXR activation	10.2	Yes
	Granulocyte adhesion and diapedesis	8.84	Yes
	Agranulocyte adhesion and diapedesis	6.21	Yes
	FXR/RXR activation	6.08	Yes
	Leukocyte extravasation signaling	5.89	Yes
	Complement system	5.05	No
	IL-8 signaling	4.98	Yes
	Coagulation system	4.89	No
	Inhibition of matrix metalloproteases	4.75	Yes
	Communication between innate and adaptive immune cells	4.65	Yes
	Extrinsic prothrombin activation pathway	4.44	No
	Glucocorticoid receptor signaling	4.02	Yes
	IL-6 signaling	3.93	Yes
	IL-10 signaling	3.74	Yes
	TREM1 signaling	3.70	Yes
	Intrinsic prothrombin activation pathway	3.68	No
	Dendritic cell maturation	3.17	No
	Paxillin signaling	3.12	Yes
	Chondroitin sulfate degradation (Metazoa)	2.89	No
	Role of cytokines in mediating communication between immune cells	2.89	Yes
	Dermatan sulfate degradation (Metazoa)	2.83	No
	Role of PRRs in recognition of bacteria and viruses	2.82	Yes
	Cytokine production in macrophages and Th cells by IL-17A/IL-17F	2.61	No
	Agrin interactions at neuromuscular junction	2.57	Yes
	Toll-like receptor signaling	2.46	Yes
	NF-κB signaling	2.28	Yes
	Crosstalk between dendritic cells and natural killer cells	2.22	Yes
	Clathrin-mediated endocytosis signaling	2.15	No
	Inhibition of angiogenesis by TSP1	2.11	Yes
	HIF1α signaling	2.07	Yes
	Oncostatin M signaling	2.06	Yes
	Integrin signaling	2.06	Yes
	IL-17A signaling in fibroblasts	2.04	Yes
	Hematopoiesis from pluripotent stem cells	1.84	No
	MSP-RON signaling pathway	1.81	Yes
	Macropinocytosis signaling	1.49	Yes
	NF-κB activation by viruses	1.43	Yes
	Integrin-linked kinase signaling	1.41	Yes
	Reelin signaling in neurons	1.37	Yes
	TR/RXR activation	1.31	No
Newborn Alum	Caveolar-mediated endocytosis signaling	6.10	Yes
	Paxillin signaling	5.55	No
	Granulocyte adhesion and diapedesis	4.67	No
	IL-8 signaling	4.49	No
	Leukocyte extravasation signaling	4.40	No
	Integrin signaling	4.38	Yes
	Crosstalk between dendritic cells and natural killer cells	4.00	No
	LXR/RXR activation	3.60	No
	Inhibition of angiogenesis by TSP1	3.31	No
	MSP-RON signaling pathway	2.99	Yes
	T helper cell differentiation	2.67	No
	Agrin interactions at neuromuscular junction	2.67	No
	TREM1 signaling	2.63	No
	NF-κB activation by viruses	2.59	No
	Reelin signaling in neurons	2.53	No
	Communication between innate and adaptive immune cells	2.49	No
	Virus entry via endocytic pathways	2.42	No
	Role of PRRs in recognition of bacteria and viruses	2.18	No
	Acute phase response signaling	1.89	No
	Dendritic cell maturation	1.89	No
	Agranulocyte adhesion and diapedesis	1.86	No
	Integrin-linked kinase signaling	1.83	No
Newborn MPLA	Granulocyte adhesion and diapedesis	8.22	Yes
	IL-8 signaling	6.52	Yes
	Leukocyte extravasation signaling	6.37	Yes
	LXR/RXR activation	6.30	Yes
	Agranulocyte adhesion and diapedesis	5.37	Yes
	Caveolar-mediated endocytosis signaling	4.53	Yes
	Acute phase response signaling	4.25	Yes
	Paxillin signaling	3.99	Yes
	IL-10 signaling	3.20	Yes
	TREM1 signaling	3.16	Yes
	Integrin signaling	2.87	Yes
	Inhibition of angiogenesis by TSP1	2.55	Yes
	Complement system	2.55	Yes
	IL-6 signaling	2.53	Yes
	Role of PRRs in recognition of bacteria and viruses	2.50	No
	FXR/RXR activation	2.44	Yes
	Role of cytokines in mediating Communication between immune cells	2.14	Yes
	Agrin interactions at neuromuscular junction	1.92	Yes
	Macropinocytosis signaling	1.91	Yes
	NF-κB activation by viruses	1.85	Yes
	Toll-like receptor signaling	1.85	Yes
	Communication between innate and adaptive immune cells	1.76	Yes
	Signaling by Rho family GTPases	1.70	No
	Virus entry via endocytic pathways	1.69	Yes
	Crosstalk between dendritic cells and natural killer cells	1.69	Yes
	Cytokine production in macrophages and Th cells by IL-17A/IL-17F	1.36	No
Newborn R848	Granulocyte adhesion and diapedesis	7.74	Yes
	IL-8 signaling	6.12	Yes
	Leukocyte extravasation signaling	5.97	Yes
	LXR/RXR activation	5.96	Yes
	Inhibition of matrix metalloproteases	5.42	Yes
	Agranulocyte adhesion and diapedesis	5.03	Yes
	Acute phase response signaling	3.97	Yes
	Paxillin signaling	3.76	Yes
	Glucocorticoid receptor signaling	3.11	Yes
	IL-10 signaling	3.03	Yes
	Macropinocytosis signaling	3.03	Yes
	TREM1 signaling	3.00	Yes
	Integrin signaling	2.66	Yes
	HIF1α signaling	2.54	Yes
	Inhibition of angiogenesis by TSP1	2.44	Yes
	Oncostatin M signaling	2.38	Yes
	IL-6 signaling	2.37	Yes
	IL-17A signaling in fibroblasts	2.36	Yes
	Role of PRRs in recognition of bacteria and viruses	2.34	Yes
	FXR/RXR activation	2.28	Yes
	MSP-RON signaling pathway	2.13	Yes
	Role of cytokines in mediating communication between immune cells	2.03	Yes
	NF-κB signaling	1.92	Yes
	Integrin-linked kinase signaling	1.84	Yes
	Agrin interactions at neuromuscular junction	1.81	Yes
	NF-κB activation by viruses	1.74	Yes
	Toll-like receptor signaling	1.74	Yes
	Reelin signaling in neurons	1.68	Yes
	Communication between innate and adaptive immune cells	1.65	Yes
	Crosstalk between dendritic cells and natural killer cells	1.58	Yes
	PTEN signaling	1.36	No

In addition to the proteins described above, we also measured vaccine-induced TNF, IL-1β and PGE_2_ release in a subgroup of 8 biological individual samples (4 newborns and 4 adults) as the *in vitro* levels of these mediators may potentially predict *in vivo* vaccine reactogenicity (21). supplemental Fig. S4 demonstrates the relationships among vaccine-induced fold changes for TNF, IL-1β, and PGE_2_ as well as LTF, PTX-3, and MMP-9. We assessed whether the *in vitro* levels of LTF, PTX-3, and MMP-9 might also predict *in vivo* vaccine reactogenicity by comparing the fold changes of each protein across two vaccine treatment groups: (1) Alum-adjuvanted HBV (Recombivax) vaccine of low reactogenicity (53) and (2) EasyFive, a combination vaccine containing whole cell pertussis vaccine of high reactogenicity (54). To assess whether a protein was differentially expressed in response to treatment with HBV (Recombivax) as compared with treatment with EasyFive, we employed the Wilcoxon rank-sum test. As shown in Table IV, EasyFive induced higher levels of positive biomarker controls TNF, IL-1β and PGE_2_ (*p* < 0.05) as compared with Recombivax, demonstrating the validity of our approach. Similarly, EasyFive also significantly induced LTF, PTX-3 and MMP-9 at the indicated fold changes as compared with Recombivax treated neonatal and adult whole blood.

**Table IV:** Alum-adjuvanted HBV (Recombivax) vs. whole cell pertussis-containing vaccine (EasyFive)

Protein	ρ value*^a^* [*number of individuals*]	
	Newborns	Adults
TNF	2.6 × 10^−5^ [*n = 4*]	1.4 × 10^−5^ [*n = 4*]
IL-1β	3.2 × 10^−5^ [*n = 4*]	3.0 × 10^−4^ [*n = 4*]
PGE_2_	3.4 × 10^−6^ [*n = 5*]	2.8 × 10^−5^ [*n = 6*]
LTF	6.2 × 10^−6^ [*n = 5*]	5.7 × 10^−5^ [*n = 5*]
PTX-3	3.6 × 10^−8^ [*n = 7*]	5.1 × 10^−7^ [*n = 8*]
MMP-9	4.6 × 10^−7^ [*n = 8*]	3.3 × 10^−7^ [*n = 8*]

*^a^* determined using Wilcoxon log-rank test.

##### Extrapolation of the Adjuvant-Induced Secretomes in vitro to Vaccine-Induced PBMC Transcriptomes In Vivo

**Fig. 7.**
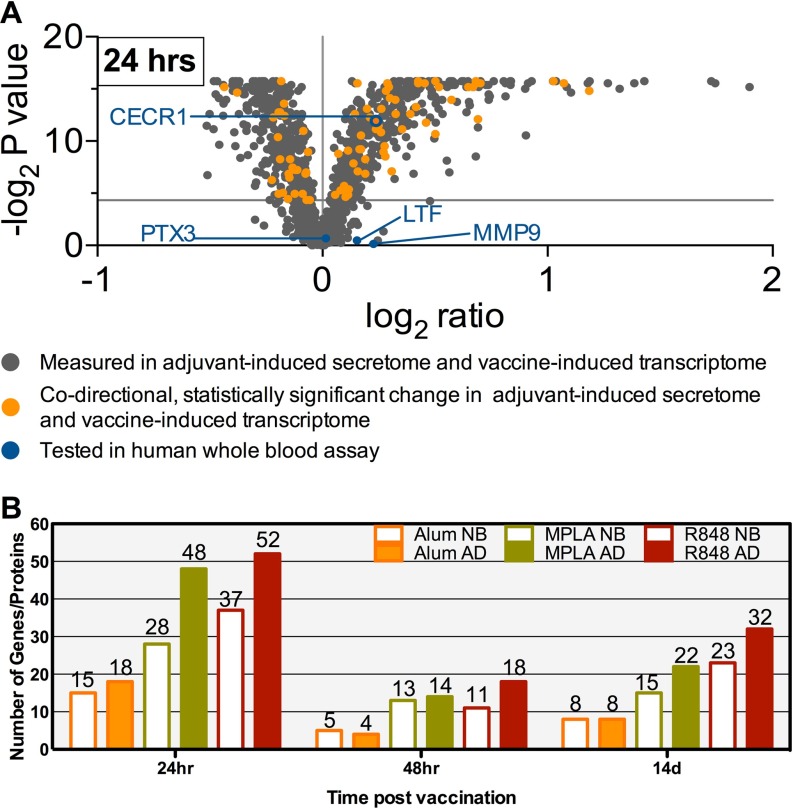
**Gene Expression Levels in Adults Immunized with an MPLA-Adjuvanted Malaria Vaccine Demonstrate Adjuvant-, Age- and Kinetic-Concordance with Secretome Proteins *in Silico*.** PBMC transcriptome profiles of 24 adults immunized with the MPLA-aduvanted Malaria vaccines RTS,S/AS01B or RTS,S/AS02A, measured at four different time points (49) were analyzed for post-immunization changes in gene expression. *A*, For each protein identified in the adjuvant-induced monocyte secretomes, the corresponding gene's fold change and *p* value at the 24 h time point was plotted on the indicated log scales. Highlighted in orange are those molecules that displayed statistically significant changes that are concordant (*i.e.* both up or both down) between vaccine-induced transcriptomes and adjuvant-induced secretomes. Blue color signifies the biomarker candidates further assessed *in vitro* in the context of this study. *B*, For each of the three post-vaccine time points, the number of molecules displaying statistically significant changes concordant between transcriptomes and newborn/adult monocyte secretomes induced by Alum and the TLR agonists MPLA and R848. Overall, this number of concordant molecules is higher for TLRA-induced secretomes than Alum-induced secretomes; higher for adult than newborn secretomes; and higher at the 24 h time point than the later time points, suggesting concordance with our *in vitro* platform. Assuming the 12,893 unique genes of the Affymetrix Human Genome U133A microarray platform used in GSE18323 as the background set of commonly measurable genes/proteins in both proteomic and transcriptomic studies, we found significant number of concordant genes between GSE18323 at 24 h with the adult secretome conditions for Alum (*p* = 4.0e-4, Kendall's tau beta test), MPLA (*p* = 1.8e-7, Kendall's tau beta test) and R848 (*p* = 2.9e-4, Kendall's tau beta) and none for the newborn data sets at 24 h. Overall, the alignment of the transcriptomic-proteomic data sets was most significant for the adult MPLA conditions.

Adjuvant-induced *in vitro* monocyte secretome data sets were compared with publicly-available *in vivo* PBMC transcriptome profiles from 24 individuals vaccinated with the MPLA-adjuvanted Malaria vaccines RTS,S/AS01B or RTS,S/AS02A (49). For each protein identified in the monocyte secretomes and represented in the transcriptome data sets, we assessed the expression profile of the corresponding gene. We found many molecules displaying statistically significant changes that were concordant (either both up or both down) between vaccine-induced transcriptomes and adjuvant-induced secretomes (Fig. 7*A* and supplemental Fig. S5). Gene ontological (GO) analysis was also performed on the *in vitro* adult MPLA-induced monocyte secretome, and each of the *in vivo* Mosquirix vaccine GSE18323 transcriptomes (24 h, 48 h and 2 weeks; supplemental Table S4), to compare the representation of gene and gene product. The GO terms significant for the MPLA secretome were compared with the GO terms significant for each transcriptome. GO terms between secretome and transcriptome overlapped at each post-vaccination time point by ∼13% (supplemental Fig. S6).

Of note, our analyses revealed that the number of the concordant (*i.e.* transcriptome *in vivo* with secretome *in vitro*) molecules appeared to correspond by adjuvant and age as it was higher for TLRA (MPLA and R848)-induced secretomes than Alum-induced secretomes; higher for adult than newborn secretomes; and higher at the 24 h time point than the later time points (Fig. 7*B*). For the purposes of comparative analysis, assuming the 12,893 unique genes of the Affymetrix Human Genome U133A microarray platform used in GSE18323 to be background set of commonly measurable genes/proteins in both proteomic and transcriptomic studies, we found significant number of concordant genes between GSE18323 at 24 h with the adult secretome conditions for Alum (*p* = 4.0e-4, Kendall's tau beta test), MPLA (*p* = 1.8e-7, Kendall's tau beta test) and R848 (*p* = 2.9e-4, Kendall's tau beta) and none for the newborn data sets at 24 h. All other pair-wise concordance analyses between the 6 secretomes and GSE18323 three time points are not significant. Of further note, the alignment of the transcriptomic-proteomic data sets was most significant for the adult MPLA conditions. This suggests that our *in vitro* model reflected age- and adjuvant-specific differences that are relevant *in vivo*.

## DISCUSSION

To our knowledge, we report the first proteomic analysis of adjuvant-induced human monocyte secretomes *in vitro*. Cluster and pathway analyses demonstrated marked differences between Alum- and TLRA-induced secretome signatures for both neonates and adults that also translated to the pathway level. Our findings are in agreement with the known functional differences between these adjuvants. Whereas TLR stimulation induces MyD88 and TRIF/TRAM dependent pathways that elicit a Th1-polarizing immune response, Alum's mode of action is distinct and still under investigation (10, 55). Alum preferentially induces a Th2 response via innate immune pathways that may include activation of the NALP3 inflammasome and of the Syk tyrosine kinase (11). Alum-induced cell necrosis releases DNA, purine catabolites and other danger-associated molecular patterns (DAMPs) that stimulate the adaptive immune response (11, 56). The ability of Alum to induce cell necrosis in our model was evident in that proteins involved in the DNA degradation pathway were induced by Alum in both newborn and adult monocytes.

Although multiple studies indicated a dampened Th1 cytokine response to LPS and to MPLA (24), suggestive of a distinct TLR4 responsiveness in newborns as compared with adults, the overall secretome response to the TLR4A MPLA in this study did not vary substantially between newborn and adult monocytes. We cannot rule out that the similarity in newborn and adult monocytes responses to TLRAs may, in part, reflect our efforts to facilitate sensitive proteomic analysis of monocytes by culturing them in the absence of autologous plasma that contains multiple soluble age-dependent factors that limit TLR-mediated neonatal Th1 cytokine production (18, 57, 58).

To further characterize adjuvant-induced proteins in a more complete and potentially more physiologic *in vitro* model, we conducted whole blood stimulation assays to confirm up-regulation of select proteins in the presence of blood plasma and multiple leukocyte types. These targeted whole blood assays confirmed that the TLRAs MPLA and R848 induced LTF, PTX-3, and MMP-9, but not ADA-2. ADA-2 may play a role in adenosine deamination in tissues or in extracellular locations with elevated levels of adenosine and low pH (48). However, it is a weak adenosine deaminase at physiological conditions and concentrations of adenosine, and is produced almost exclusively by monocyte-lineage cells. This may explain why a significant increase in ADA-2 was observed by LC/MS in the secretomes of purified monocyte secretomes, but not by a functional assay in whole blood assay supernatants.

In this same whole blood platform, we also evaluated whether conventional licensed vaccines containing the adjuvants studied may induce LTF, PTX-3, and MMP-9. Five licensed vaccines were selected for study: Recombivax, a HBV adjuvanted with Alum (amorphous aluminum hydroxyphosphate sulfate, AAHS); the HPV vaccine Cervarix, containing Alum (Al(OH)_3_) and the synthetic TLRA MPLA; PedvaxHiB, adjuvanted with Alum (AAHS) and *N. meningitidis* Outer Membrane Complex (OMPC), which stimulates TLR2 (59); Bexsero, which in addition to aluminum hydroxide contains TLR-activating *N. meningitidis* Outer Membrane Vesicles (OMVs); (60); and EasyFive, a pentavalent vaccine whose relatively high reactogenicity (propensity to induce local soreness and systemic fever after administration) is thought to be because of its whole cell pertussis vaccine component (61), known to stimulate TLR4 (60). Although the Alum-adjuvanted Recombivax induced significant up-regulation of only MMP-9 among the targeted biomarkers, all of the studied TLRA-containing vaccines induced significant LTF, PTX-3, and MMP-9 release in both age groups. We also demonstrated significantly higher levels of PTX-3 production in newborn whole blood in response to the (Alum + TLRA)-containing licensed pediatric vaccines (*i.e.* Bexsero, PedvaxHIB, and Easyfive) with relatively higher reactogenicity levels *in vivo*. In contrast, the Alum(only)-adjuvanted HBV (Recombivax), a vaccine indicated for neonatal administration with relatively low reactogenicity rates, induced higher production of MMP-9 in adult than in newborn blood.

We further scrutinized the patterns of induction of each of the targeted proteins. Whereas our proteomic (secretomic) analysis indicated significant LTF increase only for R848-stimulated neonatal monocytes, in the whole blood assay, both MPLA- (in adults) and R848- (newborns and adults) stimulation induced substantial LTF release. Robust LTF release in blood may be because of its abundance in the secondary granules of neutrophils that express functional TLRs (62). PTX-3 up-regulation following treatment with TLRA-containing vaccines is consistent with observations that PTX-3 is released in response to inflammatory stimuli, including TLRAs (63). Moreover, in a mouse model, the adjuvants MF-59 and CpG DNA (TLR9A) induce PTX-3 when injected into whole muscle *in vivo*, whereas Alum does not (29). The TLRAs MPLA and R848, as well as the TLRA-containing licensed vaccines we studied induced MMP-9, consistent with studies demonstrating TLR-mediated release of MMP-9 (64, 65). Interestingly, although Alum alone induced minimal MMP-9 release in whole blood (onefold), the Alum-adjuvanted HBV vaccine, comprised of Hepatitis B surface antigen as a viral like particle with Alum adjuvantation as well as yeast extract components that may trigger PRRs, induced a more robust response (two- to threefold). Further studies are needed to clarify the potential contribution of the Alum component of the HBV vaccine to this activity.

There is an unmet need for methods to assess candidate adjuvants with respect to their potential reactogenicity and contribution to vaccine immunogenicity and efficacy prior to clinical trials (66). Though imperfect, experimental platforms that model the human immune response to vaccines and their adjuvants *in vitro*, may recapitulate relevant aspects of the response to adjuvants and adjuvanted vaccines (1, 14, 21). Studies correlating biomarker release from human monocytic cell lines with a rabbit pyrogenicity model have suggested that immunomodulatory/inflammatory mediators such as TNF, IL-1β, or PGE_2_ may serve as *in vitro* predictors of an adjuvant's *in vivo* reactogenicity (21). We hypothesized that these proteins, as well as LTF, PTX-3, and MMP-9 could be induced in our WBA by licensed vaccines, at levels that might reflect a vaccine's empiric *in vivo* reactogenicity. Consistent with our hypothesis, the magnitude of protein biomarker induction by adjuvanted vaccines *in vitro* appeared to parallel reactogenicity *in vivo*. Alum-adjuvanted HBV (Recombivax), a vaccine of relatively low reactogenicity, induced small, if any, increases of these proteins in human WB. In contrast, marked increases of each of these proteins in human WB was noted with EasyFive, a combined formulation containing whole cell pertussis vaccine whose use was discontinued in the U.S. because of too high reactogenicity, including induction of fevers and febrile seizures (54). Further studies are needed to assess the potential utility of the proteins and pathways identified in our study as biomarkers that may correlate with vaccine reactogenicity *in vitro* and *in vivo*. If robust, such predictors could inform and accelerate development of rationally designed adjuvants and adjuvanted vaccines.

To assess whether the TLRA-induced monocyte secretome *in vitro* resembled that induced by TLRA-adjuvanted vaccine *in vivo*, we compared monocyte secretome data with published *in vivo* transcriptome data obtained from adults immunized with MPLA-containing RTS,S malaria vaccines, including the recently approved Mosquirix™ (67). Of note, we found that RTS,S-induced transcriptome changes *in vivo* mirrored proteomic (secretomic) changes in our *in vitro* platform in that alignment of gene and protein signatures was most consistent by (1) *adjuvant* with TLRA, especially MPLA-, induced secretome changes showing greater alignment than Alum; (2) *age*, as *in vivo* changes in this adult vaccine study were more similar to the adult than the newborn monocyte *in vitro* secretomes; (3) *kinetics* with *in vitro* (18 h stimulation) secretome data being more similar to the *in vivo* transcriptome at the 24 h post-vaccine than later time points; and (4) gene ontology analysis of the overlapping gene microarray transcriptome and protein monocyte secretomes for MPLA treated samples. Overall, these findings suggest a correlation between *in vitro* monocyte secretome data and *in vivo* data. We speculate that, had the *in vitro* and *in vivo* samples been derived from the same study participants, this would reduce variability related to genetics or epigenetics, likely resulting in even greater concordance of the data sets.

In conclusion, our study has for the first time employed a global proteomic approach to characterize adjuvant-induced protein release from human neonatal and adult monocytes, demonstrating adjuvant- and age-specific differences. We found that adjuvants induced different, but partially overlapping inventories of secreted proteins that varied by adjuvant type and age of study participant. TLRAs (MPLA and R848) induced secretome signatures distinct from Alum that triggered markedly different protein and pathway level responses between newborns and adults. We employed whole blood from different study participants for targeted verification (*e.g.* LTF, PTX-3, MMP-9) that proteins induced in the monocyte platform were also detectable in this more physiologically complex assay that includes autologous plasma. Finally, using publicly available adult transcriptome profiles induced by an MPLA-adjuvanted malaria vaccine, we found that the adjuvant-induced monocyte secretome *in vitro* correlated with the adjuvant-, age-, and kinetic-specific response to the same adjuvant *in vivo*. That proteins induced by pure adjuvants from monocytes *in vitro* were also induced by licensed vaccines containing the same adjuvants in whole blood *in vitro* and further verified by analysis of the transcriptomic response to an adjuvanted vaccine *in vivo*, supports their potential future utility as biomarkers for pre-clinical assessment of vaccine reactogenicity and/or immunogenicity *in vivo*. Overall, our study suggests a novel paradigm for characterizing adjuvant- and age-specific responses using age-specific *in vitro* modeling coupled with global molecular analyses and *in vivo* verification to inform rational development of age-specific adjuvanted vaccines.

## Supplementary Material

Supplemental Data

## Footnotes

^1^ The abbreviations used are:

TLR: *Toll*-like receptor
AAHS: Amorphous aluminum hydroxyphosphate sulfate
Adju-Phos: Aluminum phosphate
Alum: Aluminum phosphate, Al (PO_4_)_3_, (Adju-Phos)
APC: Antigen-presenting cell
CBMCs: Cord blood mononuclear cells
CD: Cluster of differentiation
DAMPs: Danger-associated molecular patterns
DMEM: Dulbecco's Modified Eagle Medium
DTT: dithiothreitol
ELISA: Enzyme-linked immunosorbent assay
EU: Endotoxin unit
FDR: False discovery rate
HCD: Higher-energy collisional dissociation
HPLC: High-performance liquid chromatography
HPV: Human papilloma virus
IL: Interleukin
IMQ: Imidazoquinoline
IPA: Ingenuity Pathway Analysis
LAL: *Limulus* amoebocyte lysate
LC-MS/MS: liquid chromatography coupled with tandem mass spectrometry
LPS: Lipopolysaccharide
LTF: Lactoferrin
*m*/*z*: Mass-to-charge ratio
MFI: Mean fluorescent intensity
MGF: Mascot Generic Files
MMP: Matrix metalloproteinase
Mos: Monocytes
MPLA: Monophosphoryl Lipid A
ms: Millisecond
PBMCs: Peripheral blood mononuclear cells
ppm: parts per million
PRRs: pattern recognition receptors
PTX: Pentraxin
RPMI: Roswell Park Memorial Institute Medium
SDS-PAGE: Sodium dodecyl sulfate polyacrylamide gel electrophoresis
SpC: Spectral count
TLRA: *Toll*-like receptor agonist
TNF: Tumor necrosis factor
WBA: Whole blood assay.
