# Supplementary material for: Adjuvant-induced Human Monocyte Secretome Profiles Reveal Adjuvant- and Age-specific Protein Signatures
Source: Mol Cell Proteomics. 2016 Mar 1;15(6):1877–94. doi: 10.1074/mcp.M115.055541 (PMC5083103; doi:10.1074/mcp.M115.055541)
Supplement: Supplemental Data [file supp_15_6_1877__index.html]

Adjuvant-induced human monocyte secretome profiles reveal adjuvant- and age-specific protein signatures — Adjuvant-induced Human Monocyte Secretome Profiles Reveal Adjuvant- and Age-specific Protein Signatures — Adjuvant-induced Monocyte Secretomes — Supplemental Data 

# Adjuvant-induced Human Monocyte Secretome Profiles Reveal Adjuvant- and Age-specific Protein Signatures

## Supplemental Data

- Supplemental Figures (.pdf, 2.6 MB) - Supplemental Fig. S1. Differential Protein enrichment in adjuvant-induced human monocyte secretomes (interactive). Supplemental Fig. 2. Monocyte secretomes demonstrate activation of distinct canonical pathways varying by adjuvant and age. Supplemental Fig. S3. Re-confirmation of proteins identified by monocyte secretomics using a 96-well age specific monocyte assays supplemented with autologous plasma. Supplemental Fig. S4. LTF, PTX-3 and MMP-9 in Relation to Cytokine Predictors of Vaccine Reactogenicity. Supplemental Fig. S5. Extrapolation of the Adjuvant-Induced Secretomes to Vaccine-Induced PBMC Transcriptomes. Supplemental Fig. S6. Gene ontological (GO) analysis comparing the in vitro adult MPLA-induced monocyte secretome and in vivo Mosquirix vaccine GSE18323 transcriptomes.
- Supplemental Table 1. (.xls, 814 KB) - Supplemental Table S1: Proteins detected in human monocyte secretomes
- Supplemental Table 2. (.xlsx, 165 KB) - Supplemental Table S2: Proteins significantly enriched in adjuvant-induced human monocyte secretomes.
- Supplemental Table 3. (.xlsx, 1.8 MB) - Supplemental Table S3: Protein-Identification-Information with sequence coverage.
- Supplemental Table 4. (.xls, 256 KB) - Supplemental Table S4: GO analyses were performed on the in vitro adult MPLA-induced monocyte secretome, and each of the in vivo Mosquirix vaccine GSE18323 transcriptomes (24 hrs, 48 hrs and 2 weeks; Supplemental Table S4A).
