# Supplementary material for: Adjuvant-induced Human Monocyte Secretome Profiles Reveal Adjuvant- and Age-specific Protein Signatures
Source: Mol Cell Proteomics. 2016 Mar 1;15(6):1877–94. doi: 10.1074/mcp.M115.055541 (PMC5083103; doi:10.1074/mcp.M115.055541)
Supplement: Supplemental Data [file 10.1074_M115.055541_mcp.M115.055541-1.pdf]

Supplemental Figures

Adjuvant-induced human monocyte secretome profiles reveal adjuvant- and age-specific protein signatures

Oh and Dowling, *et al*.

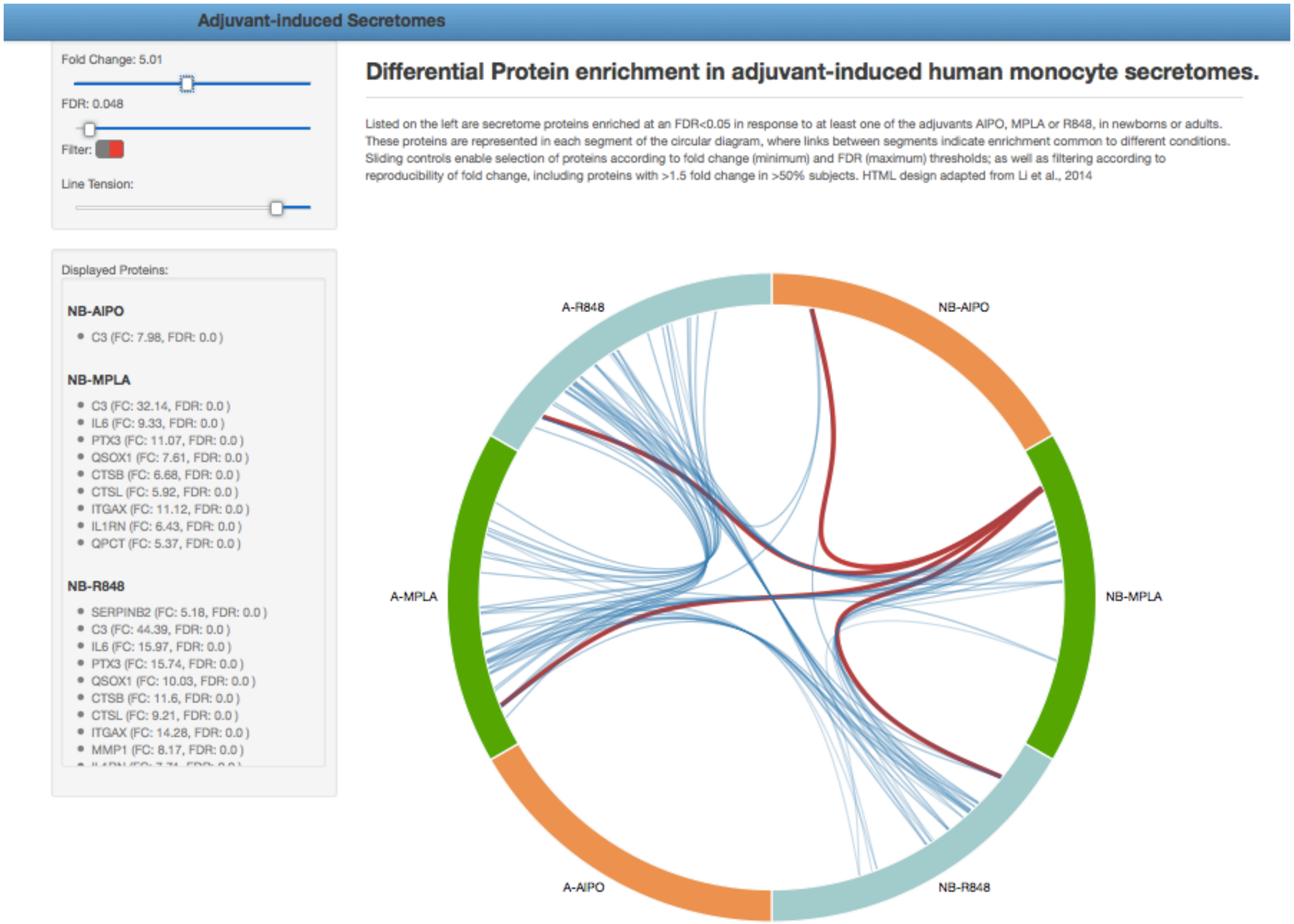

Supplemental Fig. S1. Differential Protein enrichment in adjuvant-induced human monocyte secretomes (interactive). Listed on the left are secretome proteins enriched at an FDR < 0.05 in response to at least one of the tested adjuvants (AIPO, MPLA or R848), in newborns ("NB") or adults ("A"). These proteins are represented in each segment of the circular diagram, where links between segments indicate enrichment common to different conditions. HTML design adapted from (4). The interactive version of this figure is available online at http://adjuvant-secretomes.herokuapp.com/.

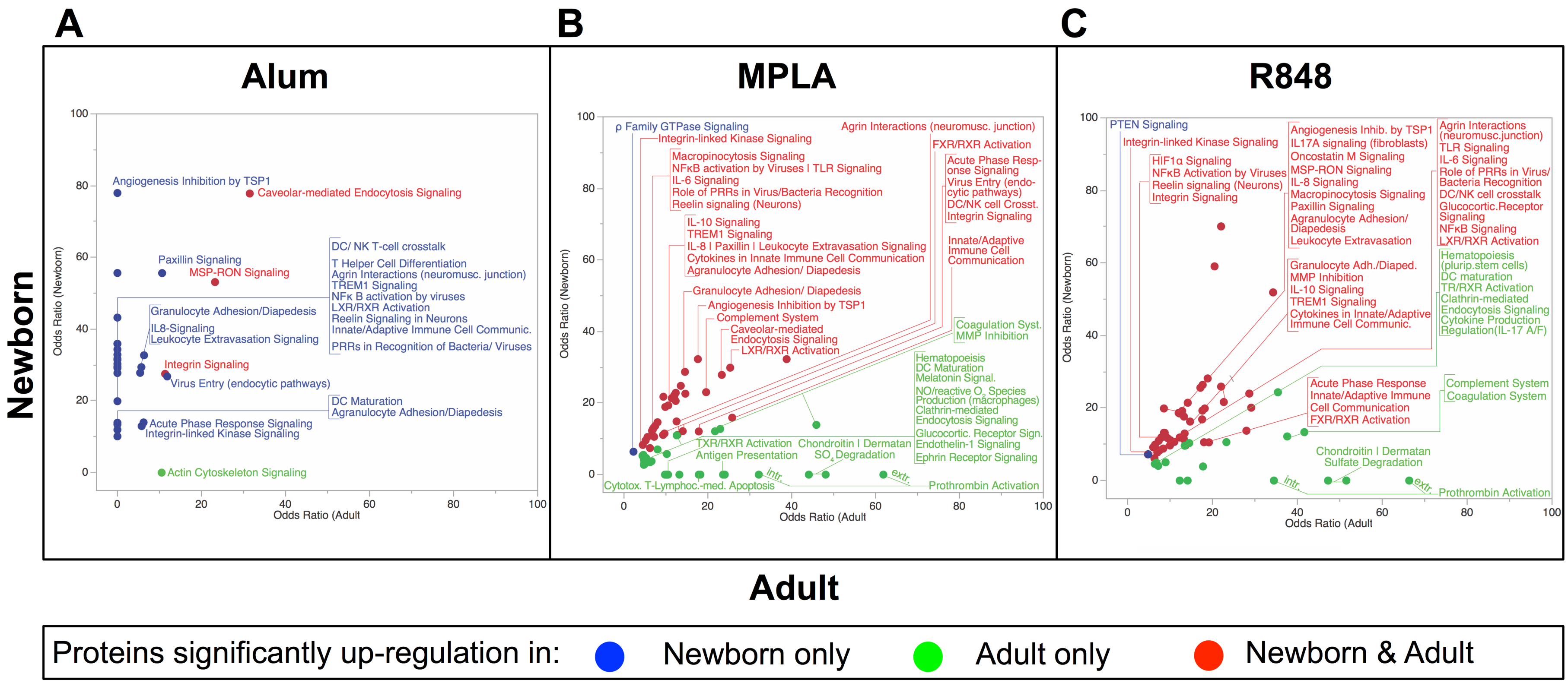

Supplemental Fig. 2. Monocyte secretomes demonstrate activation of distinct canonical pathways varying by adjuvant and age. Ingenuity Pathway Analysis (IPA) was used to identify canonical pathways represented in the sets of adjuvant-induced proteins (n = 7 newborns and n = 6 adults). Scatter plots depict, for each pathway, the Odds Ratio calculated for newborns (y-axis) and adults (x-axis) from the subsets of proteins significantly up-regulated in the A, Alum-, B, MPLA- and C, R848-induced secretomes. Interactive versions of the Scatterplots are available in the Supplemental Section online ("Fig. S1 interactive"). Color codes reflect whether a given pathway's statistically significant enrichment by IPA was observed in neonates exclusively (blue), adults exclusively (green) or both neonates and adults (red). Shown are only those pathways represented by more than one protein in at least one of the adult/ newborn datasets, with a p value < 0.05 per IPA; disease-specific pathways are excluded from the graph.

Supplemental Figure S3 Oh and Dowling, *et al*.

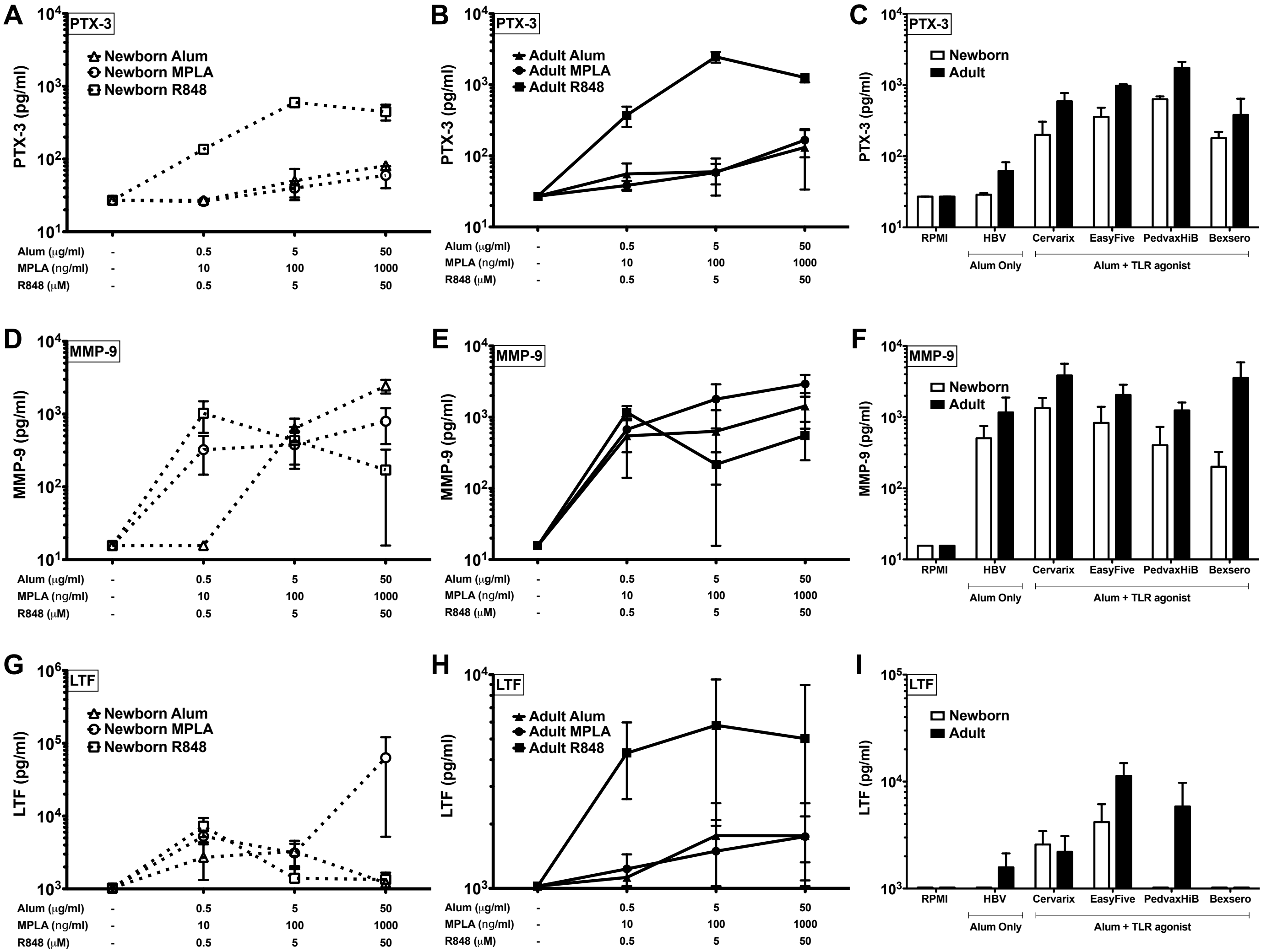

Supplemental Fig. S3. Re-confirmation of proteins identified by monocyte secretomics using a 96-well age specific monocyte assays supplemented with autologous plasma. To confirm the adjuvant and vaccine-induced release of lactoferrin (LTF), pentraxin 3 (PTX-3) and matrix metalloproteinase (MMP-9) observed in monocyte secretome assay with supplemented serum-free media, monocytes were isolated from blood using $CD14^{+}$ magnetic bead isolation and stimulated for 18 hrs in a 96-well plate supplemented with 10% autologous plasma. *A, B, D, E, G, H,* human neonatal and adult monocytes was stimulated with vehicle (RPMI), Alum (0.5 - 50 µg/ml), MPLA (TLR4; 10 - 1000 ng/ml), or R848 (TLR7/8; 0.5 - 50 µM), and supernatants were assayed for LTF, PTX-3 and MMP-9 concentrations by ELISAs. *C, F, I,* Human neonatal and adult monocytes was cultured in the presence of the vaccines Recombivax, Cervarix, EasyFive, PedvaxHIB and Bexsero. Data represents mean ± SEM after subtraction of background un-treated controls (neonates and adults; n = 3 / age group).

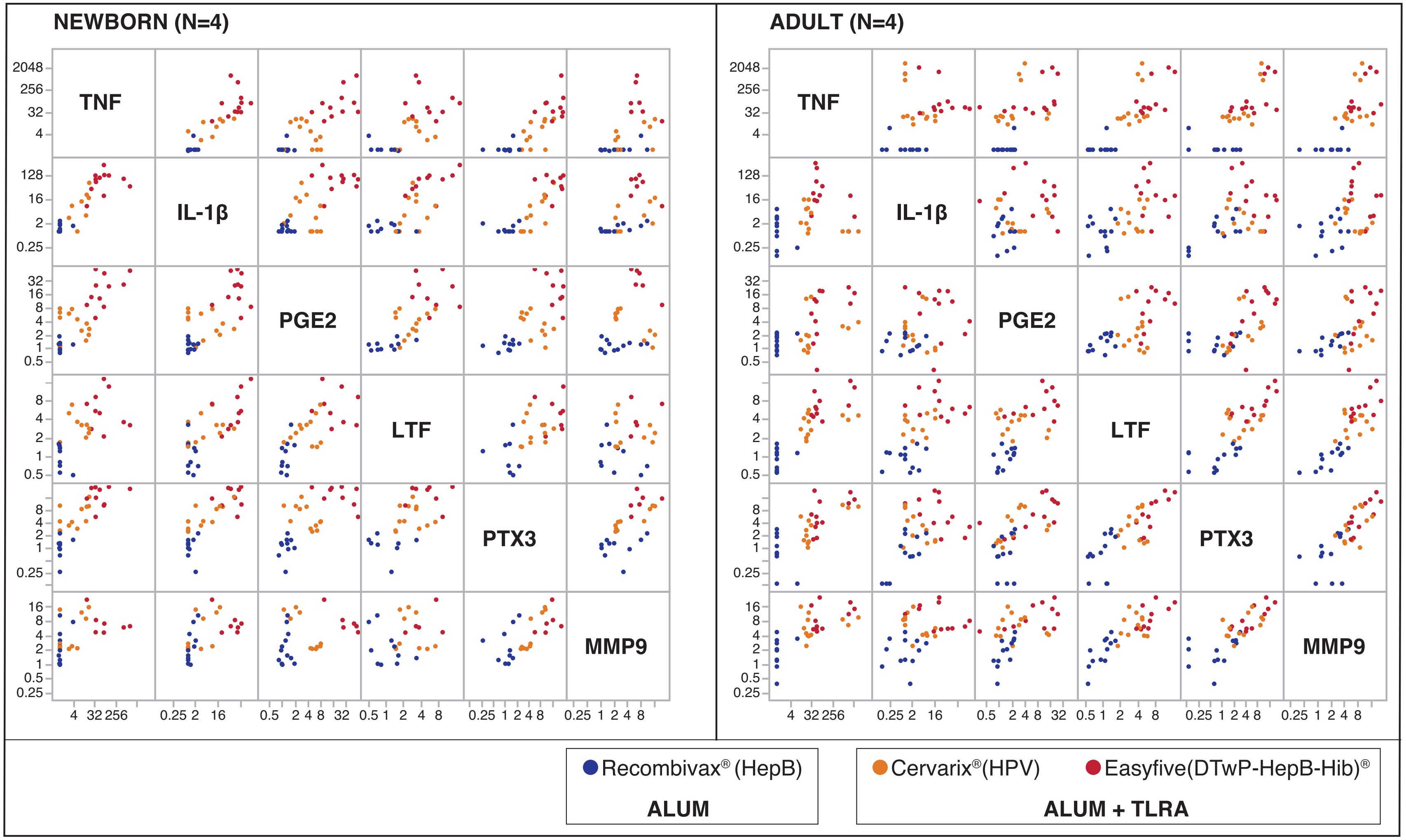

Supplemental Fig. S4. LTF, PTX-3 and MMP-9 in Relation to Cytokine Predictors of Vaccine Reactogenicity. Human neonatal and adult blood (n = 4 per age group) was cultured in the presence of the vaccines Recombivax, Cervarix and EasyFive. In the scatterplot matrix, vaccine-induced fold changes of LTF, PTX-3 and MMP-9, as well as of TNF, IL-1β and PGE_2 are plotted against each other. Vertical and horizontal axes in each scatterplot measure fold changes of those proteins indicated in the diagonal. Each dot represents the vaccine-induced fold change of the indicated protein in one blood sample at one vaccine dose; colors indicate type of vaccine. Separation of low vs. high reactogenicity vaccines was observed for multiple proteins and their combinations. (Note, the lower left triangles of Newborn / Adult scatterplot matrices are identical to the upper right triangles, with vertical/ horizontal axes symmetrically flipped.)

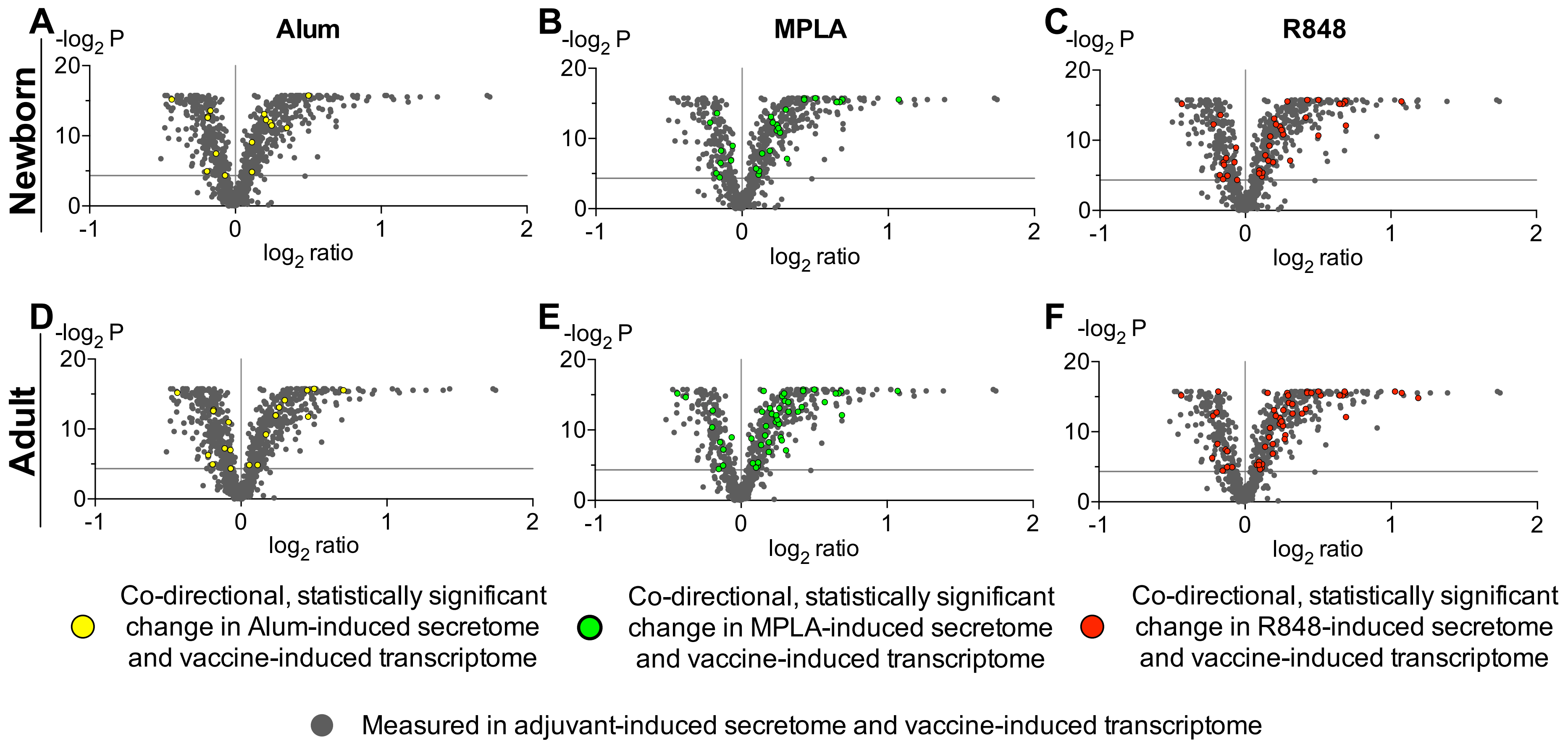

Supplemental Fig. S5. Extrapolation of the Adjuvant-Induced Secretomes to Vaccine-Induced PBMC Transcriptomes. PBMC transcriptome profiles of 24 adults immunized with the MPLA-aduvanted Malaria vaccines RTS,S/AS01B or RTS,S/AS02A, measured at 24 hr post vaccination. For each protein identified in the adjuvant-induced monocyte secretomes, the corresponding gene's fold-change and p-value at the 24 hr time point is plotted on the indicated log scales. Highlighted are those molecules that display statistically significant changes that are concordant (i.e., both up or both down) between vaccine-induced transcriptomes and adjuvant-induced newborn (*A* – *C*) or adult (*D* – *F*) monocyte secretomes induced by Alum (yellow), MPLA (green) or R848 (red).

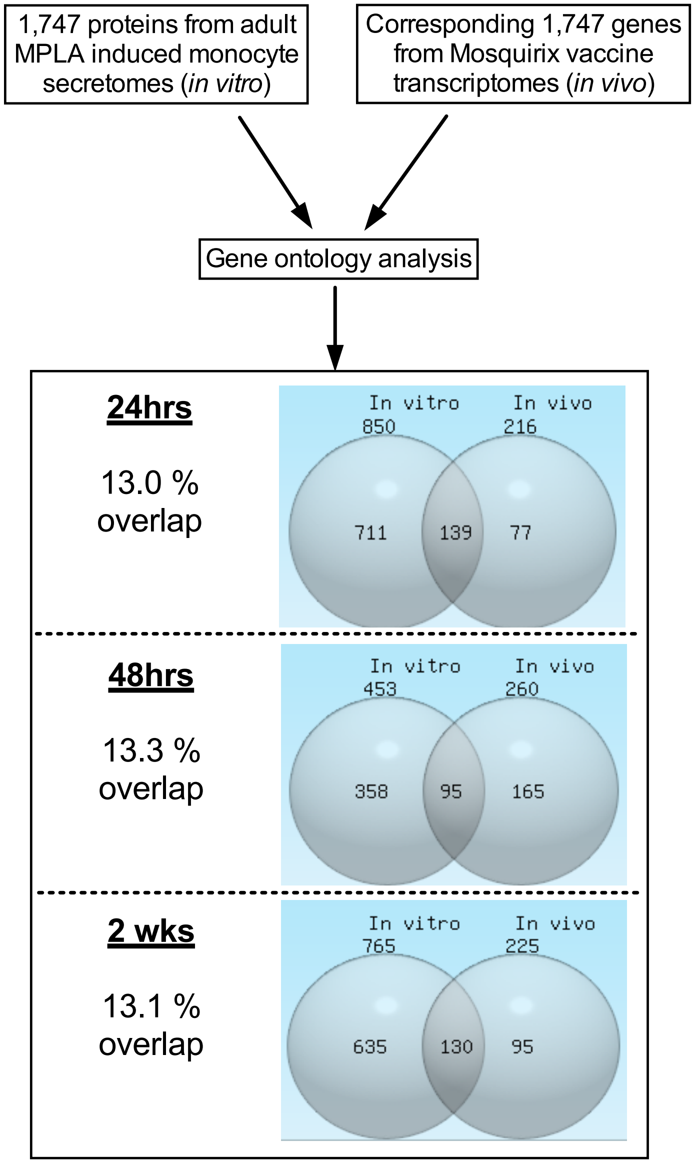

Supplemental Fig. S6. Gene ontological (GO) analysis comparing the *in vitro* adult MPLA-induced monocyte secretome and *in vivo* Mosquirix vaccine GSE18323 transcriptomes. Gene ontological analysis demonstrates the degree of overlap between the 1,747 proteins identified *in vitro* from the adult MPLA induced monocyte secretomes as compared to the corresponding *in vivo* 1,747 genes from the Mosquirix vaccine GSE18323 microarray transcriptome.
